# Metabolomic Characterization of Human Prostate Cancer Bone Metastases Reveals Increased Levels of Cholesterol

**DOI:** 10.1371/journal.pone.0014175

**Published:** 2010-12-03

**Authors:** Elin Thysell, Izabella Surowiec, Emma Hörnberg, Sead Crnalic, Anders Widmark, Annika I. Johansson, Pär Stattin, Anders Bergh, Thomas Moritz, Henrik Antti, Pernilla Wikström

**Affiliations:** 1 Department of Chemistry, Umeå University, Umeå, Sweden; 2 Department of Medical Biosciences, Umeå University, Umeå, Sweden; 3 Department of Pathology, Surgical and Perioperative Sciences, Umeå University, Umeå, Sweden; 4 Department of Urology and Andrology and Orthopedics, and Radiation Sciences, Oncology, Umeå University, Umeå, Sweden; 5 Department of Forest Genetics and Plant Physiology, Umeå Plant Science Centre, Swedish University of Agricultural Sciences, Umeå, Sweden; Baylor College of Medicine, United States of America

## Abstract

**Background:**

Metastasis to the bone is one clinically important features of prostate cancer (PCa). Current diagnostic methods cannot predict metastatic PCa at a curable stage of the disease. Identification of metabolic pathways involved in the growth of bone metastases therefore has the potential to improve PCa prognostication as well as therapy.

**Methodology/Principal Findings:**

Metabolomics was applied for the study of PCa bone metastases (n = 20) in comparison with corresponding normal bone (n = 14), and furthermore of malignant (n = 13) and benign (n = 17) prostate tissue and corresponding plasma samples obtained from patients with (n = 15) and without (n = 13) diagnosed metastases and from men with benign prostate disease (n = 30). This was done using gas chromatography-mass spectrometry for sample characterization, and chemometric bioinformatics for data analysis. Results were verified in a separate test set including metastatic and normal bone tissue from patients with other cancers (n = 7). Significant differences were found between PCa bone metastases, bone metastases of other cancers, and normal bone. Furthermore, we identified metabolites in primary tumor tissue and in plasma which were significantly associated with metastatic disease. Among the metabolites in PCa bone metastases especially cholesterol was noted. In a test set the mean cholesterol level in PCa bone metastases was 127.30 mg/g as compared to 81.06 and 35.85 mg/g in bone metastases of different origin and normal bone, respectively (P = 0.0002 and 0.001). Immunohistochemical staining of PCa bone metastases showed intense staining of the low density lipoprotein receptor and variable levels of the scavenger receptor class B type 1 and 3-hydroxy-3-methylglutaryl-coenzyme reductase in tumor epithelial cells, indicating possibilities for influx and *de novo* synthesis of cholesterol.

**Conclusions/Significance:**

We have identified metabolites associated with PCa metastasis and specifically identified high levels of cholesterol in PCa bone metastases. Based on our findings and the previous literature, this makes cholesterol a possible therapeutic target for advanced PCa.

## Introduction

Aggressive prostate cancer (PCa), eventually spreading to the bone, is a common and fatal disease requiring early diagnosis and effective treatment. Current diagnostic methods; measuring levels of prostate specific antigen (PSA) in blood samples and examining needle biopsies from the prostate under light microscopy, are however not particularly effective in separating cases of aggressive PCa from the even more prevalent and indolent forms of PCa that often can be left without treatment, or in separating cancer from other non-malignant prostate disorders [1], [2]. Using a variety of techniques multiple investigators have therefore tried to find novel diagnostic methods and prognostic markers that can separate aggressive from more indolent forms of PCa (reviewed in [3]).

Much effort has been put into the recognition of genetic and proteomic profiles for PCa (reviewed in [4]), and magnetic resonance spectroscopy have been used to exploit metabolic changes associated with PCa [5], [6], [7]. Zakian and colleauges provide a good review on the subject [8]. In a recent paper, however, Sreekumar and colleagues used liquid and gas chromatography – time of flight mass spectrometry (GC/TOFMS) to profile the metabolome in tissue, urine, and plasma from PCa patients and identified alterations associated with disease progression [9]. Specifically, they identified sarcosine, the *N*-methyl derivative of glycine, as a potentially important marker for PCa cell invasion, migration, and aggressiveness. The Sreekumar study together with other recent studies [10], [11], [12], [13] truly indicate that mass spectrometry based methods could be used to characterize metabolomic changes during cancer progression and further to identify possible diagnostic and prognostic biomarkers or biomarker patterns as well as increase our knowledge about disease progression.

This study was made with the hypothesis that potential novel markers for aggressive PCa could be discovered by finding factors markedly up-regulated in bone metastases and then examine if the same factors are also increased in blood samples and in primary tumors from patients with metastatic disease. We therefore performed a metabolomic study of PCa bone metastases in comparison with corresponding normal bone, primary PCa tumor and normal prostate tissue, using metastatic tissue collected at surgery for complications of bone metastases [14]. Furthermore, we analyzed blood samples from patients with and without diagnosed bone metastases, with the aim to identify metabolites that could be used to improve prognostication and therapy of advanced PCa. Results were verified in a separate test set also including metastatic bone tissue from other cancers. This was done using gas chromatography-mass spectrometry for sample characterization and chemometric bioinformatics for data analysis and evaluation [15].

## Results

### Prostate cancer bone metastases show clear metabolic differences to normal bone and to bone metastases from other cancers, including increased levels of cholesterol

**Figure 1:**
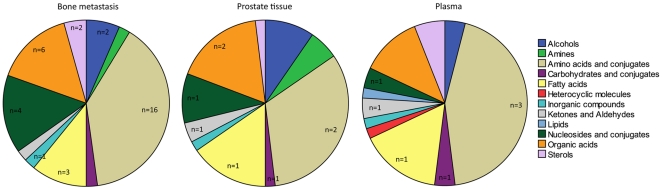
Metabolite classification. Identified metabolites are categorized according to chemical class and the number of metabolites per class significantly associated with metastasis is indicated (*P*<0.05, Mann Whitney U-test, or VIP>0.9). Classification of metabolites according to chemical class (human metabolome DB; www.hmdb.ca). n =  number of identified metabolites within each metabolite class.

**Figure 2:**
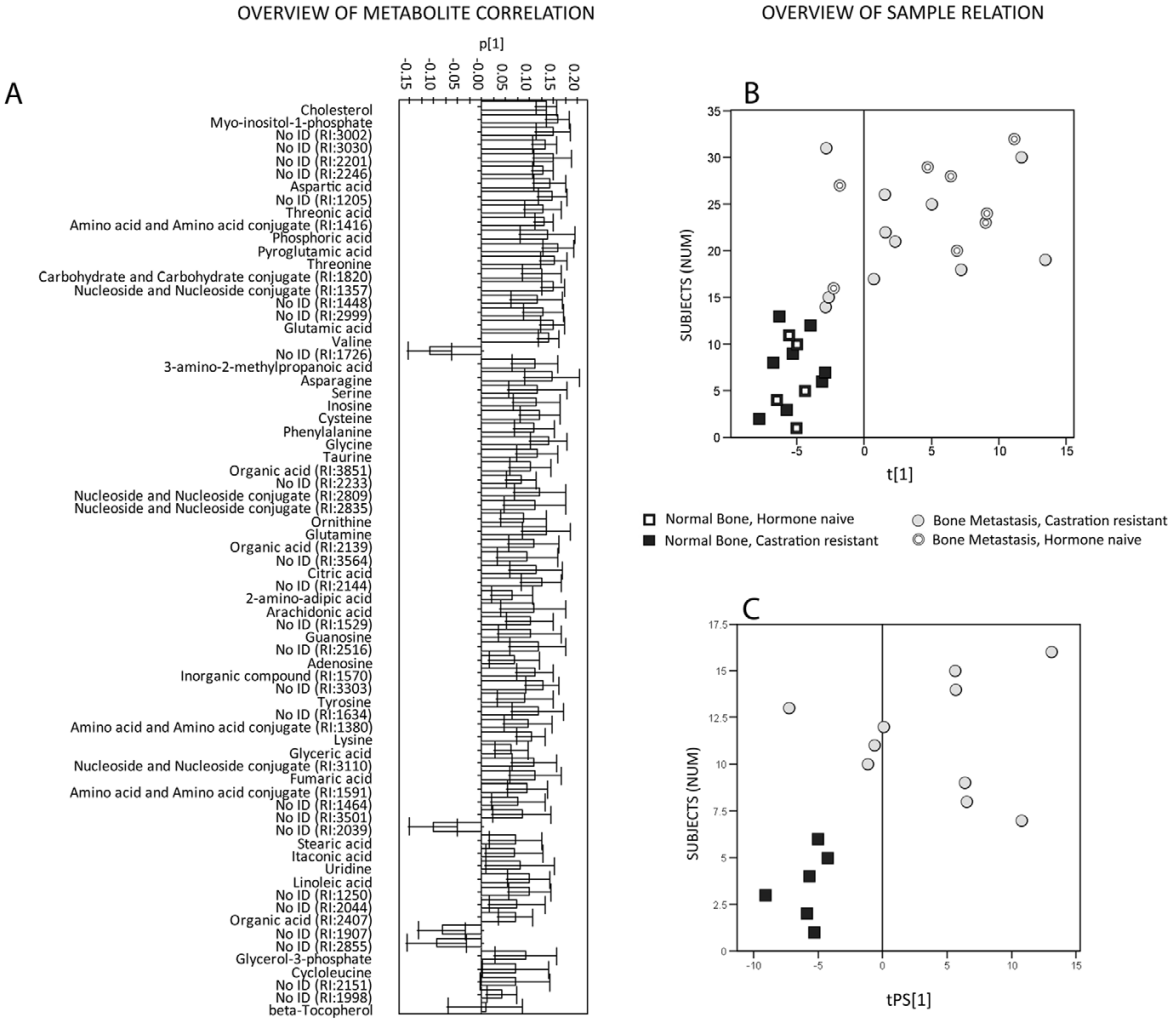
Metabolic signature in prostate cancer bone metastases. **A**) Correlation loadings (p[1]) from OPLS-DA analysis of the significantly differentiating metabolites (*P*<0.05, Mann Whitney U-test, or VIP>0.9) between prostate cancer bone metastases and normal bone showing positive values for metabolites with increased levels in bone metastases and negative values for metabolites with decreased levels in bone metastases. Classification of non-identified compounds according to chemical class (human metabolome DB; www.hmdb.ca) **B**) OPLS-DA score plot showing statistically significant separation (*P*<0.001) between normal bone and prostate cancer bone metastases. **C**) Test set predictions of prostate cancer bone metastases and corresponding normal bone samples (blind to the model) into the OPLS-DA model showing a clear discrimination between the sample classes based on the detected metabolomic signature.

Gas chromatography-time of flight mass spectrometry (GC/TOFMS) was used to characterize PCa bone metastases from 14 patients (7 hormone-naive PCa patients and 7 patients with CRPC) and adjacent normally appearing bone pieces that were available from 10 of the patients (Table 1). In total, 123 chromatographic peaks corresponding to putative metabolites were found by deconvolution [16] after exclusion of peaks originating from internal standards, contamination and artefacts. Of the 123 putative metabolites, 49 could be assigned an identity by their mass spectra and corresponding retention index (Figure 1). Orthogonal partial least squares discriminate analysis (OPLS-DA) revealed an evident and statistically significant separation (*P*<0.001) between the normal bone and bone metastasis samples, independent of treatment, determined by ANOVA of the cross-validated model (Figure 2b). Significant differences between the sample groups (VIP>0.9, Variable importance in OPLS-DA model or *P*<0.05, Mann Whitney U-test) were found for 58.5% (71 of 123) of the putative metabolites (Figure 2a, Table S1). Of the 71 significantly discriminating metabolites, 34 could be assigned an identity by their mass spectra and corresponding retention index, whereas 37 were only assigned to a possible compound class or remained unidentified. To validate the detected metabolomic signature in bone metastatic tissue and to investigate if a unique metabolite pattern for PCa exists, a set of additional samples were profiled in a separate run. This ‘test set’ included bone metastasis samples from prostate (6), breast (3), kidney (2) and squamous cell (2) adenocarcinomas as well as normal bone samples from corresponding patients prepared, profiled and predicted as a separate validation cohort (Table 1). Prediction of the PCa bone metastases and corresponding normal bone samples (blind to the model) into the OPLS-DA model revealed a clear discrimination between the sample classes in the test set (Figure 2c). In addition, a separate OPLS-DA model gave a significant difference (*P*<0.001) between the PCa bone metastases and corresponding normal bone samples in the test set and the metabolites significantly separating those sample groups (Table S2) overlapped to great extent with the significant metabolites detected in the model set (Table S1). Furthermore, OPLS-DA exposed an evident and significant (P<0.005) separation between the PCa bone metastases and the metastases from the other cancers (Figure S1).

**Table 1:** Clinical characteristics of patients operated for complications of bone metastases and included in model and test set of GC-MS analysis of bone metastases and corresponding normal bone samples.

	Metastases	Normal bone	Age (Yrs)	PSA (ng/ml)	Chemo^a^	Radiation^b^
*Model*						
**Prostate cancer**						
Hormone-naíve	7	4	79 (60–85)	130 (21–2500)	0	0
Castration-resistant^c^	7	6	69 (60–88)	690 (16–2100)	1	2
**Total no.**	14	10	73 (60–88)	160 (16–2500)	1	2
*Test set*						
**Prostate cancer**						
Castration-resistant^c^	6	4	78 (65–83)	140 (14–5139)	1	1
**Breast cancer**						
Hormone-refractory^d^	3	3	62 (43–73)	-	2	0
**Esophagus**	1	1	56	-	1	1
**Lung**	1	1	76	-		
**Kidney**	2	2	60, 82	-	1	
**Total no.**	13	11	76 (43–82)	-	5	2

*Samples are grouped according to primary diagnosis. Esophagus, lung and kidney patients were all men and breast cancer patients were women. Continuous values are given as median (min-max).*

*^a^* *Chemotherapy including estracyte, taxotere and tamoxifene prior to surgery.*

*^b^* *Radiation against operation site prior to surgery.*

*^c^* *Castration-resistant patients had disease progression after long-term androgen deprivation therapy including surgical ablation, LHRH/GNRH agonist therapy, and anti-androgen therapy; bicaglutamide.*

*^d^* *Hormone-refractory breast cancer patients had disease progression after long-term estrogen deprivation therapy including tamoxifen.*

Among the detected metabolites in PCa bone metastases (Figure 2a and Table S1) we found increased levels of several amino acids in comparison with normal bone, indicating high amino acid metabolism. Consequently, the top 12 canonical pathways in PCa bone metastases suggested by systems pathway analysis (Ingenuity Systems, Inc.) were all related to amino acid synthesis and metabolism (Table S3). Amino acid metabolism was also the top function listed by Ingenuity pathway analysis for PCa bone metastases (Table S4). Furthermore, we detected high levels of cholesterol, myo-inositol-1-phosphate, citric acid, fumarate, glycerol-3-phosphate, and fatty acids (Table S1), which are related to molecular and cellular functions within PCa bone metastases as indicated in Table S4.

**Figure 3:**
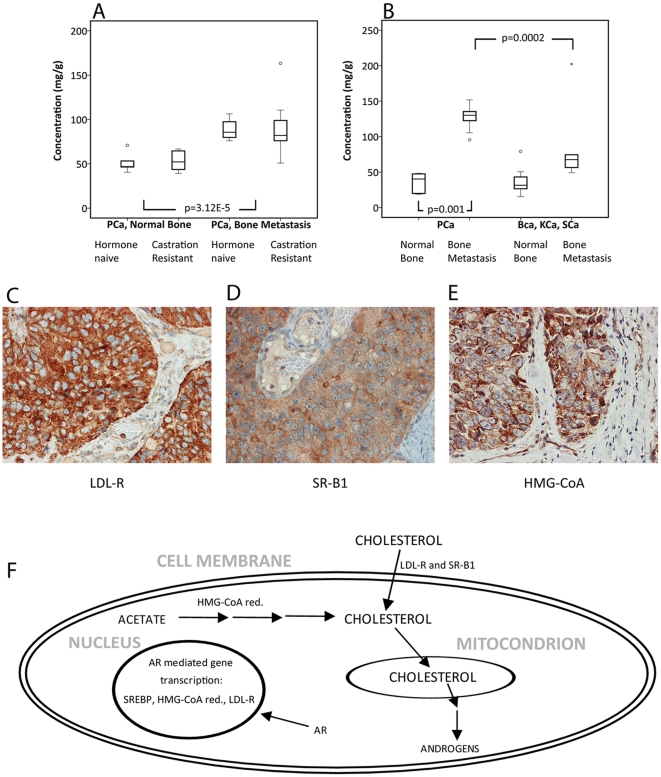
Cholesterol levels and expression of enzymes for cholesterol influx and synthesis. **A.** Box plot for cholesterol concentration (mg cholesterol/g tissue) showing significantly higher levels in prostate cancer (PCa) bone metastases compared to normal bone. **B.** Box plot for cholesterol concentration (mg cholesterol/g tissue) in test set showing significantly higher levels in PCa bone metastases compared to normal bone as well as compared to bone metastases from other cancers; breast, kidney, and squamous cancer (BCa, KCa and SCa). **C–E.** Immunohistochemical staining of the low density lipoprotein receptor (LDL-R), the scavenger receptor class B type 1 (SR-B1), and the 3-hydroxy-3-methylglutaryl-coenzyme A reductase (HMG-CoA red.) in PCa bone metastases showing intense staining and indicating possibilities for influx as well as *de novo* synthesis of cholesterol in tumor epithelial cells, as suggested in F. **F.** Cholesterol influx and synthesis is stimulated by androgen receptor (AR) action partly via activation of the sterol regulatory element-binding protein (SREBP) and subsequent transcription of LDL-R and HMG-CoA red [24], [25] and androgens could be provided from cholesterol by its conversion in several steps [27], [28].

We specifically noted the high levels of cholesterol in PCa bone metastases as cholesterol showed the highest VIP value when differentiating PCa bone metastases from normal bone tissue (Table S1, Figure 2a) as well as from other bone metastases (Table S5). Moreover, cholesterol has been suggested to promote cancer development and progression (reviewed in [17]), and was therefore chosen for further analysis. The high cholesterol level in PCa bone metastases as compared to normal bone (*P* = 3.12E-5, Figure 3a) was clearly verified in the test set data (*P* = 0.001, Figure 3b). Interestingly, the cholesterol levels in PCa bone metastases were high also in comparison with levels in bone metastases from other cancers (P = 0.0002, Figure 3b).

### Prostate cancer bone metastases have the machinery for uptake and *de novo* synthesis of cholesterol

Cholesterol constitutes a potential therapeutic target and we therefore wanted to examine possible reasons to the high cholesterol levels in PCa bone metastases. Cells can obtain cholesterol by exogenous influx via the low density lipoprotein receptor (LDL-R), the scavenger receptor class B type 1 (SR-B1) or by *de novo* synthesis from acetyl-CoA where the reduction of 3-hydroxy-3-methylglutaryl-coenzyme A (HMG-CoA) into mevalonate is considered to be the rate-limiting step [18], [19]. Paraffin-embedded pieces from the PCa metastases included in the GC/TOFMS analysis were therefore immunostained for LDL-R, SR-B1 and HMG-CoA reductase. All PCa cases showed strong homogeneous LDL-R staining of metastatic epithelial cells and occasionally less intense staining of adjacent stroma cells, endothelial cells, adipocytes, and bone cells (Figure 3c, Table 2). The epithelial staining of SR-B1 and HMG-CoA reductase was more heterogeneous, ranging from weak to intense (Table 2). Interestingly, HMG-CoA reductase showed particularly intense staining in endothelial cells, vessel walls, immune cells, and bone cells (Figure 3e), while SR-B1 showed negative stroma staining (Figure 3d). Our results show that tumor epithelial cells in PCa bone metastases possibly synthesize cholesterol *de novo* via HMG-CoA reductase, but also that other cell types in the bone metastases micro-environment express this enzyme possibly allowing them to provide cholesterol that could be taken up by tumor epithelial cells through the LDL and SRB-1 receptors (Figure 3f). There was no obvious relation between the heterogeneity in SR-B1 and HMG-CoA immunostaining and the corresponding cholesterol levels in the PCa bone metastases (data not shown). Interestingly, however, the PCa bone metastases showed generally stronger immunostaining of LDL-R and SR-B1 than the bone metastases of different origin (Table 2), possibly contributing to the relatively higher cholesterol levels seen in the PCa metastases (Fig. 3).

**Table 2:** Immunohistochemical staining of LDL-R, SR-B1, and HMG-CoA reductase in prostate cancer bone metastases and in bone metastasis of different origin.

Bone metastases	n	LDL-R n (%)		SR-B1 n (%)			HMG-CoA red. n (%)	
		+	++	-	+	++	+	++
**Prostate cancer**								
Hormone-naíve	7		7 (100)		5 (71)	2 (29)	2 (29)	5 (71)
Castration-resistant	13		13 (100)		7 (54)	6 (46)	8 (62)	5 (38)
**Total**	**20**		**20 (100)** **		**12 (60)**	**8 (40)** **	**10 (50)**	**10 (50)**
**Different origin** a								
**Total**	**7**	**4 (57)**	**3 (43)**	**4 (57)**	**2 (29)**	**1 (14)**	**4 (57)**	**3 (43)**

*^a^* *Samples were grouped according to metastasis origin; prostate cancer or other origin. For further clinical characteristics and treatments, please see Table 1.*

^**^ *Higher fraction with intensely (++) stained cells as compared with metastases of different origin, P<0.01. For definition of staining intensity, please see Materials and Methods.*

### Metabolic differences between primary prostate tumor tissues from high-risk patients with and without established bone metastases

Primary PCa tissue obtained from patients with high-risk tumors (defined as locally advanced or poorly differentiated cancer; stage T3-4 and/or GS 8-10) with (M1, n = 7) or without (M0, n = 6) diagnosed bone metastases were profiled in comparison with benign prostate samples (n = 17, Table 3). This resulted in 157 putative metabolite peaks of which 59 could be assigned an identity (Figure 1). Clear and statistical significant discrimination (P<0.001), determined by ANOVA of the cross-validated model, between all three prostate tissue classes (benign, M0, and M1) was revealed by OPLS-DA modelling (Figure S2). Significant changes associated with metastatic disease, defined as metabolite changes in M1 vs. benign and M1 vs. M0 (P<0.05 or VIP>0.9) were detected for 13 metabolites of which eight were identified (Tables S6 and S7). Interestingly, four of those were also significantly increased in bone metastases samples as compared to normal bone; aspargine, threonine, fumaric acid, and linoleic acid (Table S1).

**Table 3:** Clinical characteristics of patients included in GC-MS analysis of plasma and corresponding prostate biopsy samples.

	Plasma			Biopsy^b^		
	M1^a^	M0	Benign	M1^a^	M0	Benign
**GS** c						
*6*	2	4			1	
*7*	2	3		7	3	
*8-10*	11	6			2	
**T** d						
*T1*	4	2				
*T2*	1	1		1	1	
*T3*	10	10		6	5	
**PSA** e (ng/ml)	37 (4.8–997)	16 (9.9–173)	9.0 (4.2–56)	40 (4.8–695)	17 (14–71)	8.9 (4.2–56)
**Age** f (yrs)	68 (48–86)	68 (58–80)	66 (51–77)	68 (61–85)	66 (58–77)	65 (59–77)
**Total**	15	13	30	7	6	17

*Continuous values are given as median (min-max).*

*^a^* *According to bone scan.*

*^b^* *No cancer diagnosis or high grade prostate interneoplasia at biopsy.*

*^c^* *Tumor differentiation according to Gleason score (GS).*

*^d^* *Clinical tumor stage according to Union Internationale Contre le Cancer.*

*^e^* *Serum PSA at date of blood draw and prostate biopsy.*

*^f^* *Age at date of blood draw and prostate biopsy.*

### Distinguishing metabolite profiles in blood plasma from patients with high risk tumors with and without established bone metastases

Investigation of the plasma metabolome from PCa patients with (M1, n = 15) and without (M0, n = 13) diagnosed bone metastases and men with benign disease was based on 179 resolved putative metabolites, and of those 50 could be assigned an identity (Figure 1). Despite the clear overlap in serum PSA levels (Table 3), a significant separation (P<0.003) using OPLS-DA modelling was obtained for the difference between M1 and benign plasma as well as between M1 and M0 plasma, determined by ANOVA of the cross validated models. Twenty-seven metabolites, seven identified, were found as significantly altered (P<0.05 or VIP>0.9) in the blood plasma from M1 patients in comparison with patients with benign (Table S8) and M0 disease (Table S9). Interestingly, of these 27, four metabolites; glutamic acid, taurine, and phenylalanine (elevated in the blood) and stearic acid (decreased in blood) were also found as metastasis markers in bone (Table S1). A summary of all identified metabolites in the different sample types are given in Table S10 and data can be found in a supporting data file (Data S1).

### Sarcosine levels in tissue and plasma samples

Sarcosine levels were measured separately in the samples using AccQ•Tag derivatization followed by LC/MS analysis. The analysis showed an increase of sarcosine in PCa bone metastases compared to normal bone, while no difference could be observed compared to bone metastases from other cancers (Figure S3). In addition, no clear disease progression was seen when comparing the sarcosine levels between benign prostate and primary prostate tumor tissue, although the low number (n = 5) of primary tumor extracts available for this analysis did affect the reliability of the results, and also made a comparison between primary tumor and bone metastasis tissues unreliable. Comparisons of sarcosine levels in the blood plasma revealed no significant differences related to PCa or the presence of bone metastases (data not shown).

## Discussion

We here, for the first time, report a comprehensive analysis of metabolic patterns in PCa bone metastases in comparison to primary PCa, benign prostate tissue, and normal bone tissue. We have found metabolites which differentiate PCa bone metastases from normal bone samples and, furthermore, from bone metastases of different origin. We also found metabolites which, in contrast to PSA, showed altered plasma and primary tumor levels in individuals with metastatic PCa in comparison with patients with high-risk tumors but without detectable metastases. One of our most notable findings is high levels of cholesterol in the PCa bone metastases, which is probably reached by *de novo* synthesis of cholesterol in tumor epithelial cells as well as influx of this metabolite from the surroundings via LDL-R and SR-B1.

Increased bioavailability of cholesterol in tumor cells may have high biological relevance for bone metastases growth, as cholesterol supplementation has been shown to increase PCa tumor cell proliferation, migration, and invasion *in vitro* [20] while cholesterol targeting induces apoptosis [21], probably by lowering lipid raft cholesterol content and thereby interfering with growth factor signalling [21], [22]. The prostate gland normally contains high levels of cholesterol in comparison to other organs and increased cholesterol levels have previously been associated with PCa [23]. Further elevation of cholesterol levels in bone metastases could obviously reflect high demand for membrane biosynthesis in proliferating cells, but also the fact that cholesterol metabolism is directly regulated by androgens (reviewed in [24]). Androgens positively regulate LDL-R gene transcription and also promote cholesterol synthesis by increasing transcription of HMG-CoA reductase [25] and thus the rate-limiting conversion of HMG-CoA to mevalonate [26]. As androgen receptors are expressed and presumingly active in a majority of bone metastases in CRPC [14], androgen actions probably contribute to the higher cholesterol levels in PCa metastases as compared to bone metastases of different origin. Cholesterol in turn possibly contributes to androgen receptor signalling and thus castration resistant tumor growth in patients treated with androgen-deprivation therapy, by its conversion into androgens by metabolic enzymes [27], [28]. Accordingly, a Western diet and high serum levels of cholesterol have been associated with increased PCa risk in a number of studies [29], [30], [31], although the results have not been completely conclusive (reviewed in [17]). Interestingly, a recent study shows a lower risk of developing high-grade PCa for men with low serum cholesterol levels [32] and, in line with this, long-term use of HMG-CoA reductase inhibitors (“statins”) for prevention of cardiovascular disease have been shown to reduce the risk of PCa progression into aggressive, fatal disease [33], [34], [35], [36]. Cholesterol-lowering agents have also been shown to inhibit growth of PCa cells *in vitro* and in model experimental systems *in vivo* [37], [38]. Taken together, those results indicate the possibility of using cholesterol inhibitors as treatment or chemopreventive agents for PCa metastasis, but novel drugs are then needed as the statins used today primarily concentrate to the liver and poorly reach peripheral organs [17].

We found high levels of many amino acids within the PCa bone metastases, and amino acid metabolism was the most altered functional pathway associated with PCa bone metastases according to Ingenuity pathway analysis. Our results thus support the metabolomic-based study by Sreekumar and co-workers [9] and also earlier gene-expression-based studies showing increased protein synthesis during PCa progression [39]. Interestingly, a recent paper high-lighted that also levels of aminoacyl tRNA synthetases (aaRSs) are increased during PCa progression and, furthermore, that transcription of some aaRSs is stimulated by androgens [40]. This remarkable finding could possibly be connected to the fact that we found increased levels of certain amino acids such as threonine, glutamate, phenylalanine within PCa bone metastases in comparison with bone metastases of different origin. In addition to the amino acids, other notable metabolites in our data (citric acid, fumarate, glycerol-3-phosphate, and fatty acids) indicate a high energy metabolism that could reflect the high fraction of proliferating cell within bone metastases [14]. Moreover, the high levels of myo-inositol-1-phosphate could be a sign of active cell signalling involving inositol-based molecules as second messengers, such as inositol phosphates and phosphatidylinositol phosphates. Those molecules are involved in activating protein kinase C and Akt and thus in regulating processes considered as hallmarks of cancer, i.e. cell proliferation, apoptosis, differentiation, invasion, and angiogenesis [41]. Overall the alterations in the metabolome detected in this study as associated with PCa bone metastases indicated disturbed molecular and cellular functions of clear relevance for cancer progression. The relative importance of those functions are however difficult to assign, as they partly depend on the metabolite classes detectable within the GC/TOFMS analysis and the identities obtained within currently available libraries. With the GC/TOFMS metabolite profiling method we were not able to detect sarcosine in the samples. However, using a targeted analysis approach, we found high levels of sarcosine in bone metastases in accordance with previous findings of increased levels of sarcosine with PCa progression [9]. Importantly though, we did not see any difference in sarcosine levels between PCa bone metastases and bone metastases of different origin, indicating that sarcosine is not PCa specific but instead associated with advanced cancer and metastasis. A more comprehensive picture of specific biological networks of importance for PCa metastases growth will be obtained as analysis methods develop and libraries for identification get more complete, but could also be achieved by combining metabolomic data with genomic or proteomic data. So far, we specifically note that threonine, aspargine, fumaric acid, and linoleic acid are increased not only in bone metastases but also in primary prostate tissue from patients with confirmed bone metastases when compared with M0 patients. Essential fatty acid such as linoleic acid have been shown to stimulate PCa tumor growth in model systems [42], [43], and the conversion of linoleic acid to arachidonic acid and further into prostaglandins could possibly stimulate an inflammatory response which is associated with pathogenesis of PCa [44].

Neither cholesterol nor sarcosine were, however, prognostic for bone metastases in plasma. Instead high levels of glutamatic acid, phenylalanine, and taurine were found in PCa bone metastasis tissue and in plasma from men with diagnosed PCa bone metastases. Glutamic acid was recently shown in the study by Sreekumar and collegues to be increased in PCa tissue [9] and, interestingly, cancer cells that cause bone disruption in animal models secrete glutamate into their environment [45]. As glutamatergic intercellular communication is important for normal bone homeostasis via glutamate receptors on specific bone cells (reviewed in [46]) it is possible that disturbances within this system could be detected during the process of bone metastasis. Our results are also in line with a recent study that found higher taurine levels in PCa than in benign tissue when assessed using magic angle spinning ((1)H HR-MAS) NMR spectroscopy [47]. The value of these metabolites as plasma markers for aggressive PCa however needs to be confirmed in further studies.

In conclusion, we have identified metabolites associated with prostate cancer metastasis and specifically noted high levels of cholesterol in PCa bone metastases. Based on our findings and the previous literature, this makes cholesterol a possible therapeutic target for advanced PCa. Although this is the largest metabolomic study of PCa bone metastases performed it certainly has its limitations. Previous ^1^H NMR studies have revealed evident changes in levels of citrate and choline between benign prostate and tumor tissue [5], [6], [7] but somewhat concerning, we were not able to find such differences in citrate levels. Neither could we with our method detect choline. These results do highlight limitations of our method and point out the need for complementary approaches in the search for useful metabolomic biomarkers. Furthermore, the rather low number of patients included and the heterogeneity of metastatic disease make evaluation in further studies necessary before the significance of our results could be assured.

## Materials and Methods

### Ethics statement

Studies were approved by the local ethic review board of Umeå University and participants gave written or verbal consent.

### Samples

Bone metastases and adjacent normally appearing bone tissue pieces were obtained from a series of fresh-frozen biopsies collected from patients with cancer diagnosis or suspicion of cancer, operated for metastatic spinal cord compression or pathologic fractures (Table 1). Patients have been thoroughly described in [14].

Blood plasma was available from a series of men who underwent transrectal ultrasound–guided needle biopsies of the prostate, due to increased serum PSA levels, and primary PCa and benign prostate biopsies were assessable in some cases (Table 3). The patients included in this study were all selected to have high-risk tumors defined as; presence of bone metastases or a locally advanced tumor or a poorly differentiated cancer (M1 and/or T3-4 and/or GS 8-10), while men with benign disease had at least two rounds of negative biopsies. The PCa cases and the benign cases were matched according to time since sampling. Additional information about patients and sample preparation is given in supporting text (Text S1).

### Metabolomic profiling using GC/TOFMS

Prior to GC/TOFMS analysis the low molecular weight metabolites in plasma samples were extracted and derivatized as previously described [48]. Tissue samples were extracted with H_2_O/methanol/chloroform (1∶3∶1) mixture containing 11 internal standards A [48] (1 mL per 15 mg of tissue) evenly distributed over the chromatographic retention span. Extraction was carried on in bead mill with two tungsten beads and the rest of the procedure was the same as for plasma samples. Derivatized sample extracts were then injected in splitless mode by an CTC Combi Pal autosampler (CTC Analytics AG, Zwingen, Switzerland) into an Agilent 6890 gas chromatograph equipped with a 10 m×0.18 mm i.d. fused silica capillary column with a chemically bonded 0.18 µm DB 5-MS stationary phase (J&W Scientific, Folsom, CA, USA). The column effluent was introduced into the ion source of a Pegasus III time-of-flight mass spectrometer, GC/TOFMS (Leco Corp., St Joseph, MI, USA). An alkane serie (C10-C40) was run for each separate GC/TOFMS run. More details regarding sample preparation, derivatization and GC/TOFMS analyses can be found in the supplementary information. The reproducibility of the method has been reported previously [16], [48].

### Data processing

Data pre-treatment including baseline correction, chromatogram alignment, time-window setting, hierarchical multivariate curve resolution (H-MCR) [11] and normalization were performed in MATLAB [version 7.3] using custom scripts. More details regarding the data processing can be found in the supporting information (Text S1).

### Data analysis and Statistics

Orthogonal partial least squares - discriminant analysis (OPLS-DA) [49] was applied to extract and interpret the systematic variation in the resolved GC/TOFMS tissue and plasma profiles related to specific responses. The objective was to extract metabolic patterns related to PCa and more specifically to metastatic disease. The OPLS-DA variable importance in the projection (VIP) values combined with univariate p-values (Mann-Whitney U-test) were used to highlight significant metabolites or metabolite patterns (VIP>0.9 or *P*<0.05). ANOVA of the cross-validated models were used to determine the significance of the extracted metabolite patterns. Comparisons of categorical data were made using the chi-square test. For more details see supporting information (Text S1).

### Pathway analysis

Differentiating metabolites between PCa bone metastases and normal bone (VIP>0.9 in OPLS-DA and/or *P*<0.05 in Mann-Whitney U-test, were included in pathway analysis according to Ingenuity Systems, Inc. Top altered canonical pathways and cellular and molecular functions in bone metastases were listed.

### Metabolite identification

Detected peaks were identified by a data base search, based on spectra and chromatographic retention index, using NIST MS-Search v. 2.0 [50] with the in-house mass spectra library database established by Umeå Plant Science Center (UPSC), the mass spectra library maintained by the Max Planck Institute in Golm (http://csbdb.mpimp-golm.mpg.de/csbdb/gmd/gmd.html) or the NIST98 mass spectra library. For a more detailed description, see supporting text (Text S1).

### Immunohistochemistry

Samples were fixed in buffered formalin, decalcified in formic acid, and embedded in paraffin. Five µm paraffin-embedded sections were de-paraffinated and rehydrated according to standard procedures and boiled in 10 mM Tris, 1 mM EDTA (pH = 9) for 1 h in 2100 Retriever (HistoLab, Frölunda, Sweden). Primary antibody incubations and secondary systems were as follows; Anti-LDL receptor (ab52818, Abcam, Cambridge, UK, diluted 1∶250) and Anti-SRB-1 (EP1556Y, Novus Biologicals, Littleton, CO, diluted 1∶50) were visualized using the iVIEW™ DAB detection kit (Ventana, Tucson, AZ) while Anti-HMG-CoA reductase (Upstate, cat 07-457, Millipore, Temecula, CA, diluted 1∶250) were incubated over night and further detected using the ABC Vectastain kit (Vector Laboratories, Burlingame, CA) with DAB as chromogen. The staining intensity was scored as weak (+) or intense (++) and for SR-B1 also as negative (-) in a few cases. Positive controls for the immunostaining assays (liver and ovarian tissues) showed strong staining and control sections that were incubated without primary antibodies showed no staining.

## Supporting Information

Text S1 (0.04 MB DOC)

Table S1 (0.20 MB DOC)

Table S2 (0.09 MB DOC)

Table S3 (0.04 MB DOC)

Table S4 (0.03 MB DOC)

Table S5 (0.07 MB DOC)

Table S6 (0.07 MB DOC)

Table S7 (0.07 MB DOC)

Table S8 (0.06 MB DOC)

Table S9 (0.07 MB DOC)

Table S10 (0.10 MB DOC)

Figure S1 Multivariate modelling in the search for a unique metabolite pattern in prostate cancer (PCa) bone metastases. OPLS-DA score vector (t[1]) showing a clear and significant difference between PCa bone metastases and bone metastases from other cancers: breast, kidney, and squamous cancer (BCa, KCa and SCa).
(2.24 MB TIF)

Figure S2 Metabolomic differences between primary prostate cancer tissues from high-risk patients with (M1) and without (M0) established bone metastases. OPLS-DA score plot (t[2]/t[1]) revealing clear differences in metabolic signatures in primary prostate tumor tissue from patients with and without diagnosed bone metastases compared to benign prostate tissue.
(2.40 MB TIF)

Figure S3 Sarcosine levels in bone metastases. A. Box plot for sarcosine concentration showing significantly higher levels in prostate cancer (PCa) bone metastases compared to normal bone. B. Box plot for sarcosine in test set showing higher levels in PCa bone metastases compared to normal bone but no difference in levels in PCa bone metastases compared to bone metastases from other cancers; breast, kidney, and squamous cancer (BCa, KCa and SCa).
(4.93 MB TIF)

Data S1 (0.38 MB XLS)
