# Supplementary material for: Metabolomic Characterization of Human Prostate Cancer Bone Metastases Reveals Increased Levels of Cholesterol
Source: PLoS One. 2010 Dec 3;5(12):e14175. doi: 10.1371/journal.pone.0014175 (PMC2997052; doi:10.1371/journal.pone.0014175)
Supplement: Text S1 — (0.04 MB DOC) [file pone.0014175.s001.doc]

**Supporting Text:**

**Materials and Methods**

Patients

Fresh-frozen biopsies from bone metastases and adjacent normal bone were collected at the Dept of Orthopedics (Umeå University Hospital) from patients operated for metastatic spinal cord compression or pathologic fractures between Sep. 2003 and July 2009. Plasma and fresh-frozen prostate biopsies were collected at the Dept of Urology (Umeå University Hospital) from patients who underwent transrectal ultrasound–guided needle biopsies of the prostate, due to increased serum levels PSA between Sept 2003 and July 2008. Data on primary tumor characteristics were obtained from the National Prostate Cancer Register, Northern Sweden part, Oncological Centre at Umeå University Hospital, and included tumor-node-metastasis system classification and tumor differentiation (Gleason score, GS) (Table 3). Clinical characteristics of patients operated for complications of bone metastasis were obtained by reviewing medical record (Table 1).

Tissue and plasma preparation

Presence or absence of tumor cells in the tissue samples was confirmed by light microscopy of hematoxylin/eosin-stained tissue sections and the fraction of tumor cells (ranging from 20 to 90 %) were estimated by one pathologist. Representative areas of bone metastases, normal bone, primary prostate cancer and benign tissue were cut with a sterile scalpel, weighted, and put into a 1.5 ml extraction tube (Sarstedt). Blood plasma was drawn into one EDTA tube, shortly before the needle biopsies were taken, stored for 30 min in RT and plasma was collected after centrifugation in 1500 g for 10 min, snap-frozen in liquid nitrogen and stored in -80°C.

Preparation of tissue and plasma samples for GC/TOFMS analysis

Just before extraction plasma samples were allowed to thaw at 37^o^C for 15 min. To 100 µL of plasma, 900 µL of extraction solution (methanol/water (8:1) with 11 internal standards [48] (concentration of each equal 7 ng μl^-1^), evenly distributed over the entire retention time span, was added and samples were vigorously extracted at a frequency of 30 Hz for 2 min using a MM301 vibration Mill (Retsch GmbH & Co. KG, Haan, Germany). After 120 min on ice, the samples were centrifuged at 19600 g for 15 min at 4°C. A 200 µL aliquot of supernatant was transferred to a GC vial and evaporated to dryness.

Tissue samples were kept in liquid nitrogen until addition of extraction solution, which was H_2_O/methanol/chloroform (1:3:1) mixture (1 mL per 15 mg of tissue) containing the same 11 internal standards as for the plasma samples. Extraction was carried out using a MM301 vibration Mill (Retsch GmbH & Co. KG, Haan, Germany) with two tungsten beads at a frequency of 30 Hz for 2 min. After removal of beads, samples were centrifuged at 19600 g for 15 min at 4°C. A 200 µL aliquot of supernatant was transferred to a GC vial and evaporated to dryness. Methoxymation of dried extracts with 30 µL of methoxyamine solution in pyridine (15 μg μL^-1^) was carried out at room temperature for 16 h. Then samples were trimethylsilylated with 30 µL of MSTFA at room temperature for 1 h, after which 30 µL of heptane (containing 0.5 µg of methyl stearate as an injection internal standard) were added.

Metabolite profiling with GC/TOFMS

A 1 µL aliquot of derivatized sample was injected splitless by a CTC Combi Pal autosampler (CTC Analytics AG, Zwingen, Switzerland) into an Agilent 6980 GC equipped with a 10 m x 0.18 mm i.d. fused-silica capillary column chemically bonded with 0.18 µm DB5-MS stationary phase (J&W Scientific, Folsom, CA). The injector temperature was set at 270°C. Helium was used as carrier gas at a constant flow rate of 1 mL min-1 through the column. For every analysis, the purge time was set to 60 s at a purge flow rate of 20 mL min-1 and an equilibration time of 1 min. The column temperature was initially kept at 70°C for 2 min and then increased from 70 to 320°C at 30°C min-1, where it was held for 2 min. The column effluent was introduced into the ion source of a Pegasus III TOFMS (Leco Corp., St Joseph, MI). The transfer line temperature was set at 250°C and ion source temperature at 200°C. Ions were generated by a 70 eV electron beam at a current of 2.0 mA. Masses were acquired from m/z 50 to 800 at a rate of 30 spectra s-1, and the acceleration voltage was turned on after a solvent delay of 155 s. An alkane serie (C_10_-C_40_) was run for each separate GC/TOFMS run.

Quantification of cholesterol was done using the corresponding internal standard [2H7]-cholesterol as a reference.

Sarcosine quantification

AccQ•Tag derivatization of sarcosine was performed according to the manufacturer’s protocol. 200ul of dried extract was resuspended in 20uL of 20mM HCl and 60uL of AccQ•Tag Ultra borate buffer was added to each vial. Finally 20uL of the freshly prepared AccQ•Tag derivatization solution was added and the sample was immediately vortexed for 10s. After mixing the sample were let standing for 30 minutes in room temperature followed by 10 minutes at 55°C.

Liquid chromatography was performed on a 1200 series binary pump system from Agilent Technologies. The chromatographic separation was performed on a Hypersil Gold column (50x2.1 mm, 1.9 µm particle size) from Thermo Scientific; the column temperature was 55°C and the sample injection volume was 2 µL. Aqueous formic acid (0.1 %) was used as eluent A whereas eluent B was acetonitrile with 0.1 % formic acid. The mobile phase rate was 0.5 mL/min throughout the analysis. Before start of the analysis the column was equilibrated with 0.1 % B. The HPLC gradient was as follows: the initial condition was 0.1 % B and from 0.54 to 8 minutes the B eluent was linearly increased from 0.1 % to 6 %. At 8 minutes the percentage of B eluent was linearly increased to reach 95 % at 10 minutes and eluent B was kept at 95 % until 11 minutes. From 11 to 12 minutes the column was returned to its initial conditions (0.1 % B). The column was equilibrated for 4 minutes before injection of the subsequent sample. The mass spectrometry detection was performed on a 6460 Triple quadruple LC/MS from Agilent Technologies. The mass spectrometer, equipped with a Jetstream electrospray source (ESI), was run in positive mode. The source temperature was 325°C, the sheath gas flow and temperature was 12 L/h and 400°C, respectively, and the capillary voltage was held at 4 kV. The MS/MS analysis was conducted using multiple reaction monitoring (MRM) mode. The MRM conditions for sarcosine and the internal standard were optimized using MassHunter MS Optimizer software (Aglient Technologies). For sarcosine the selected precursor ion was 260.2 m/z, the quantifier product ion was 171 m/z, and for fragmentation the fragmentor voltage was 86 V and the collision energy 35 V. For true peak identity another mrm transition was performed for sarcosine using m/z 260.2 as precursor ion, and m/z 182.9 as a qualifier product ion. The fragmentor voltage and collision energy were 86 V and 35 V respectively. Glutamic acid labeled with stable isotopes was used as internal standard. The mrm transition for the internal standard was: precursor ion 324.1 m/z and product ion m/z 171. The optimized fragmentation conditions were fragmentor voltage 109 V and collision energy 22 V.

The detected peak areas were integrated using Masshunter Quantitative Analysis software (Agilent technologies).

Data processing using hierarchical multivariate curve resolution

Nonprocessed GC-MS data files were exported from the instrument in common data format (.cdf). Alignment and smoothing using a moving average was done prior to dividing the chromatograms into time windows, from which chromatographic profiles (peaks representing putative derivatized metabolites) with corresponding mass spectra were resolved by an alternating regression procedure for each time window separately. Prior to multivariate statistical analysis, normalization of chromatographic peak areas was performed using 11 internal standards eluting over the entire chromatographic time range.

Data analysis

Orthogonal partial least squares - discriminant analysis (OPLS-DA) was applied to compare samples based on their metabolite profiles. Cross validation and test set predictions (bone metastasis model) was used to establish the predictive ability of the models. ANOVA calculated from the cross-validated OPLS-DA score vectors (CV-ANOVA) was used to obtain p-values for the detected differences between pre-defined sample classes in the respective models. The OPLS-DA variable importance in the projection (VIP) values combined with univariate *P* -values (Mann-Whitney U-test) were used to highlight significant metabolites or metabolite patterns (VIP > 0.9 or *P* < 0.05). All multivariate statistical analyses were performed in the SIMCA-P+ software version 12.5 (Umetrics AB, Umeå, Sweden), while univariate statistical tests were carried out in SPSS 17.0 software (SPSS Inc., Chicago, IL).

Metabolite identification

Detected peaks were identified by a spectral data base search, based on spectral fragmentation pattern and chromatographic retention index, using NIST MS-Search v. 2.0 (15), using the in-house mass spectra library database established by Umeå Plant Science Center (UPSC), the mass spectra library maintained by the Max Planck Institute in Golm (<http://csbdb.mpimp-golm.mpg.de/csbdb/gmd/gmd.html>), or the NIST98 mass spectra library. Match values ranking the spectra were calculated using the dot product of the two spectra (i.e. the resolved spectrum and the database spectrum), with higher m/z peaks having more weight than lower m/z peaks, since higher m/z values are considered to be more compound specific. This included a reverse logic ignoring "impurity" (i.e. non-matching) peaks in the resolved spectrum, which is not penalized for peaks in the target spectrum that are not present in the database spectrum. Match values range from 0-999, with 999 being an identical match. Positive identification verification was obtained by combining match values from the data base search with retention time index, calculated based on the analytically characterized alkane series (C_10_-C_40_).
