# Supplementary material for: Metabolomic Characterization of Human Prostate Cancer Bone Metastases Reveals Increased Levels of Cholesterol
Source: PLoS One. 2010 Dec 3;5(12):e14175. doi: 10.1371/journal.pone.0014175 (PMC2997052; doi:10.1371/journal.pone.0014175)
Supplement: Table S1 — (0.20 MB DOC) [file pone.0014175.s002.doc]

**Table S1.** Differentiating metabolites between prostate cancer (PCa) bone metastases and normal bone (NB) tissue in relation to metastasis-associated metabolites^*^ in primary prostate tissue and in plasma as well as in bone metastasis of different origin.

| **METABOLITE** | **BONE METASTASIS** | | **BONE METASTASIS TESTSET** | | | | **PROSTATE TISSUE** | | | | **PLASMA** | | | |
| --- | --- | --- | --- | --- | --- | --- | --- | --- | --- | --- | --- | --- | --- | --- |
|  | PCa vs. NB | | PCa vs. NB | | PCa vs. Ca | | M1 vs. M0 | | M1 vs. Benign | | M1 vs. M0 | | M1 vs. Benign | |
| Cholesterol | ↑ | <0.001 | ↑ | 0.001 | ↑ | <0.001 |  | 0.366 | ↑ | 0.247 |  | 0.359 |  | 0.322 |
| Myo-inositol-1-phosphate | ↑ | <0.001 | ↑ | 0.002 | ↑ | 0.033 |  | 0.199 | ↑ | 0.035 | ↑ | 0.176 |  | 0.765 |
| No ID (RI:3002) | ↑ | <0.001 | ↑ | 0.001 | ↑ | 0.020 |  | 0.245 | ↑ | 0.203 |  | 0.15 | ↑ | 0.056 |
| No ID (RI:3030) | ↑ | <0.001 | ↑ | 0.001 | ↑ | 0.002 |  | 0.063 |  | 0.116 |  |  |  |  |
| No ID (RI:2201) | ↑ | <0.001 | ↑ | 0.002 | ↑ | 0.016 |  | 0.317 | ↑ | 0.018 |  |  |  |  |
| No ID (RI:2246) | ↑ | <0.001 |  |  |  |  |  |  |  |  |  |  |  |  |
| Aspartic acid | ↑ | <0.001 | ↑ | 0.010 |  | 0.556 |  | 0.519 | ↑ | 0.064 |  | 0.275 |  | 0.845 |
| No ID (RI:1205) | ↑ | <0.001 |  |  |  |  |  | 0.418 | ↑ | 0.083 | ↑ | 0.021 | ↑ | 0.036 |
| Threonic acid | ↑ | <0.001 | ↑ | 0.002 | ↑ | 0.001 |  | 0.487 | ↑ | 0.037 | ↑ | 0.251 |  | 0.181 |
| Amino acid and Amino acid conjugate (RI:1416) | ↑ | <0.001 |  |  |  |  | ↓ | 0.011 |  | 0.563 |  | 0.295 |  | 0.322 |
| Phosphoric acid | ↑ | <0.001 | ↑ | 0.002 | ↑ | 0.137 |  |  | ↑ | 0.001 |  | 0.631 |  | 0.908 |
| Pyroglutamic acid | ↑ | <0.001 | ↑ | 0.004 | ↑ | 0.042 |  |  | ↑ | 0.004 | ↑ | 0.009 |  | 0.205 |
| Threonine* | ↑ | <0.001 | ↑ | 0.007 | ↑ | 0.003 | ↑ | 0.032 | ↑ | 0.064 |  | 0.783 |  | 0.698 |
| Carbohydrate and Carbohydrate conjugate (RI:1820) | ↑ | <0.001 | ↑ | 0.004 | ↑ | 0.004 |  |  |  |  |  |  |  |  |
| Nucleoside and Nucleoside conjugate (RI:1357) | ↑ | <0.001 |  |  |  |  |  | 0.116 | ↑ | 0.565 |  |  |  |  |
| No ID (RI:1448) | ↑ | 0.001 |  | 0.366 |  | 0.790 | ↓ | 0.165 | ↑ | 0.008 | ↓ | 0.089 |  | 0.591 |
| No ID (RI:2999) | ↑ | <0.001 |  |  |  |  |  |  |  |  |  | 0.15 | ↑ | 0.056 |
| Glutamic acid* | ↑ | <0.001 | ↑ | 0.005 | ↑ | 0.039 |  | 1.00 | ↑ | 0.008 | ↑ | 0.15 | ↑ | 0.134 |
| Valine | ↑ | 0.001 | ↑ | 0.014 | ↑ | 0.006 |  | 0.439 | ↑ | 0.105 | ↑ | 0.335 |  | 0.593 |
| No ID (RI:1726) | ↓ | <0.001 |  | 0.606 |  | 0.934 | ↓ | 0.085 |  | 0.277 |  |  |  |  |
| 3-amino-2-methylpropanoic acid | ↑ | <0.001 |  |  |  |  |  |  |  |  |  |  |  |  |
| Asparagine* | ↑ | <0.001 | ↑ | 0.007 |  | 0.424 | ↑ | 0.003 | ↑ | 0.105 |  | 0.6 |  | 0.695 |
| Serine | ↑ | <0.001 | ↑ | 0.007 | ↑ | 0.003 | ↑ | 0.022 |  | 0.225 |  | 0.793 |  | 0.908 |
| Inosine | ↑ | <0.001 | ↑ | 0.007 | ↑ | 0.021 |  | 0.38 |  | 0.298 |  |  |  |  |
| Cysteine | ↑ | <0.001 | ↑ | 0.003 | ↑ | 0.004 |  | 0.817 |  | 0.418 |  | 0.748 |  | 0.382 |
| Phenylalanine* | ↑ | <0.001 | ↑ | 0.053 | ↑ | 0.001 | ↑ | 0.005 |  | 0.165 | ↑ | 0.013 | ↑ | 0.065 |
| Glycine | ↑ | <0.001 | ↑ | 0.001 |  | 0.191 |  | 0.132 | ↑ | 0.011 | ↑ | 0.141 |  | 0.884 |
| Taurine* | ↑ | 0.004 | ↑ | 0.039 | ↑ | 0.062 |  | 0.643 | ↑ | 0.02 | ↑ | 0.222 | ↑ | 0.069 |
| Organic acid (RI:3851) | ↑ | <0.001 |  |  |  |  |  | 0.225 | ↑ | 0.277 |  |  |  |  |
| No ID (RI:2233) | ↑ | <0.001 |  |  |  |  |  | 0.643 | ↑ | 0.003 |  |  |  |  |
| Nucleoside and Nucleoside conjugate (RI:2809) | ↑ | <0.001 | ↑ | 0.002 | ↑ | 0.051 |  | 0.848 | ↑ | 0.048 |  |  |  |  |
| Nucleoside and Nucleoside conjugate (RI:2835) | ↑ | <0.001 |  |  |  |  |  |  |  |  |  |  |  |  |
| Ornithine | ↑ | <0.001 | ↑ | 0.007 | ↑ | 0.011 |  | 0.817 |  | 0.427 | ↑ | 0.174 |  | 1.000 |
| Glutamine | ↑ | 0.003 | ↑ | 0.016 | ↑ | 0.021 |  | 0.848 | ↑ | 0.085 |  | 0.275 |  | 0.596 |
| Organic acid (RI:2139) | ↑ | 0.002 |  |  |  |  |  |  |  |  | ↓ | 0.168 | ↓ | 0.125 |
| No ID (RI:3564) | ↑ | 0.003 |  |  |  |  | ↓ | 0.132 | ↑ | 0.132 |  |  |  |  |
| Citric acid | ↑ | <0.001 | ↑ | 0.001 | ↑ | 0 |  | 0.643 |  | 0.38 |  | 0.854 |  | 0.607 |
| No ID (RI:2144) | ↑ | <0.001 |  |  |  |  |  |  |  |  |  |  |  |  |
| 2-amino-adipic acid | ↑ | 0.005 | ↑ | 0.025 | ↑ | 0.009 |  | 0.655 | ↑ | 0.018 |  |  |  |  |
| Arachidonic acid | ↑ | <0.001 | ↑ | 0.002 |  | 0.155 |  | 0.897 | ↓ | 0.083 |  | 0.793 |  | 0.836 |
| No ID (RI:1529) | ↑ | 0.016 | ↑ | 0.015 | ↑ | 0.033 |  | 0.796 | ↑ | 0.021 |  |  |  |  |
| Guanosine | ↑ | <0.001 |  |  |  |  | ↓ | 0.203 | ↑ | 0.093 |  |  |  |  |
| No ID (RI:2516) | ↑ | 0.003 |  |  |  |  |  | 0.439 |  | 0.728 |  |  |  |  |
| Adenosine | ↑ | <0.001 | ↑ | 0.005 | ↑ | 0.007 | ↓ | 0.302 |  | 0.908 |  |  |  |  |
| Inorganic compound (RI:1570) | ↑ | 0.006 | ↑ | 0.004 |  | 0.186 |  | 0.105 | ↑ | 0.003 |  |  |  |  |
| No ID (RI:3303) | ↑ | 0.016 |  |  |  |  |  |  |  |  |  |  |  |  |
| Tyrosine | ↑ | <0.001 | ↑ | 0.005 | ↑ | 0.002 | ↑ | 0.021 |  | 0.247 | ↑ | 0.108 |  | 0.332 |
| No ID (RI:1634) | ↑ | <0.001 |  |  |  |  |  |  |  |  |  |  |  |  |
| Amino acid and Amino acid conjugate (RI:1380)* | ↑ | 0.004 | ↑ | 0.005 | ↑ | 0.006 |  | 0.253 | ↑ | 0.035 |  |  |  |  |
| Lysine | ↑ | 0.005 | ↑ | 0.013 | ↑ | 0.006 |  | 0.487 | ↑ | 0.083 |  | 0.407 |  | 0.626 |
| Glyceric acid | ↑ | 0.023 | ↑ | 0.002 | ↑ | 0.058 | ↑ | 0.015 |  | 0.728 |  | 0.631 |  | 0.312 |
| Nucleoside and Nucleoside conjugate (RI:3110) | ↑ | 0.004 |  |  |  |  |  |  |  |  |  |  |  |  |
| Fumaric acid* | ↑ | 0.013 | ↑ | 0.002 | ↑ | 0.001 | ↑ | 0.004 | ↑ | 0.003 |  |  |  |  |
| Amino acid and Amino acid conjugate (RI:1591)* | ↑ | 0.025 | ↑ | 0.003 | ↑ | 0.005 |  |  |  |  |  |  |  |  |
| No ID (RI:1464) | ↑ | 0.006 |  | 0.242 |  | 0.248 |  | 0.247 | ↑ | 0.011 |  | 0.57 |  | 0.661 |
| No ID (RI:3501) | ↑ | 0.016 | ↑ | 0.013 | ↑ | 0.003 | ↓ | 0.062 | ↑ | 0.176 |  |  |  |  |
| No ID (RI:2039) | ↓ | 0.031 |  |  |  |  |  |  |  |  |  |  |  |  |
| Stearic acid* | ↑ | 0.005 | ↑ | 0.020 |  | 0.248 |  | 0.728 |  | 0.908 | ↓ | 0.19 | ↓ | 0.093 |
| Itaconic acid | ↑ | 0.054 |  |  |  |  | ↓ | 0.355 |  | 0.165 |  |  |  |  |
| Uridine | ↑ | <0.001 | ↑ | 0.003 |  | 0.248 |  | 0.063 |  | 0.18 |  |  |  |  |
| Linoleic acid* | ↑ | 0.020 | ↑ | 0.007 | ↑ | 0.008 | ↑ | 0.01 | ↑ | 0.11 |  | 0.176 |  | 0.322 |
| No ID (RI:1250) | ↑ | 0.027 |  |  |  |  |  |  |  |  |  |  |  |  |
| No ID (RI:2044) | ↑ | <0.001 |  |  |  |  |  |  |  |  |  |  |  |  |
| Organic acid (RI:2407) | ↑ | 0.002 |  | 0.366 |  | 0.248 |  | 0.317 |  | 0.949 |  |  |  |  |
| No ID (RI:1907) | ↓ | 0.044 |  |  |  |  |  |  |  |  |  | 0.76 |  | 0.696 |
| No ID (RI:2855) | ↓ | 0.041 |  |  |  |  |  |  |  |  |  |  |  |  |
| Glycerol-3-phosphate | ↑ | 0.016 | ↑ | 0.032 |  | 0.094 |  | 0.225 | ↑ | 0.013 |  |  |  |  |
| No ID (RI:1361) | ↑ | 0.014 |  |  |  |  |  |  |  |  |  |  |  |  |
| No ID (RI:2151) | ↑ | 0.003 |  |  |  |  |  |  |  |  |  |  |  |  |
| No ID (RI:1998) | ↑ | 0.034 |  | 0.156 | ↑ | 0.013 |  | 0.699 | ↑ | 0.064 |  |  |  |  |
| beta-Tocopherol | ↑ | 0.015 |  |  |  |  |  |  |  |  |  |  |  |  |

Significant changes defined as VIP > 0.9 in OPLS-DA or *P* < 0.05, Mann Whitney U-test, indicated with arrow. A non-significant *P*-value is shown to indicate that the metabolite was detected in the analysis.

RI = Retention Index. ^*^Significant changes (P < 0.05 or VIP > 0.9) associated with metastatic disease were defined as metabolite changes found in comparisons of patients with diagnosed bone metastases (M1) to patients with benign disease and to high-risk patients without detectable metastases (M0) in plasma or prostate tissue samples (Tables S6-9).
