# Supplementary material for: Metabolomic Characterization of Human Prostate Cancer Bone Metastases Reveals Increased Levels of Cholesterol
Source: PLoS One. 2010 Dec 3;5(12):e14175. doi: 10.1371/journal.pone.0014175 (PMC2997052; doi:10.1371/journal.pone.0014175)
Supplement: Table S2 — (0.09 MB DOC) [file pone.0014175.s003.doc]

**Table S2.** Significantly differentiating metabolites between prostate cancer (PCa) bone metastases and normal bone (NB) in test set.

| **Metabolite** | **p-value** | **Increase/Decrease PCa vs. NB** |
| --- | --- | --- |
| myo-Inositol-1-phosphate* | 0.002 | ↑ |
| Ribose | 0.002 | ↑ |
| Phosphoric acid* | 0.002 | ↑ |
| No ID (RI:2203)* | 0.002 | ↑ |
| Cholesterol* | 0.001 | ↑ |
| Threonic acid* | 0.002 | ↑ |
| No ID (RI:3033)* | 0.001 | ↑ |
| Arachidonic acid | 0.002 | ↑ |
| No ID (RI:1752) | 0.003 | ↑ |
| Proline | 0.001 | ↑ |
| Uridine* | 0.002 | ↑ |
| Uracil | 0.002 | ↑ |
| Glycine | 0.001 | ↑ |
| No ID (RI:1482)* | 0.003 | ↑ |
| Nucleoside and Nucleoside conjugate (RI:2817)* | 0.002 | ↑ |
| Fumaric acid* | 0.002 | ↑ |
| Pyroglutamic acid* | 0.004 | ↑ |
| No ID (RI:3701)* | 0.002 | ↑ |
| Carbohydrate and Carbohydrate conjugate (RI:1871) | 0.002 | ↑ |
| No ID (RI:3006)* | 0.001 | ↑ |
| No ID (RI:1675)* | 0.005 | ↑ |
| Adenosine-5-monophosphate | 0.001 | ↑ |
| Glyceric acid* | 0.002 | ↑ |
| Guanine* | 0.001 | ↑ |
| No ID (RI:1990) | 0.003 | ↑ |
| Uridine* | 0.003 | ↑ |
| No ID (RI:1596)* | 0.003 | ↑ |
| No ID (RI:1382)* | 0.005 | ↑ |
| Glutamic acid* | 0.005 | ↑ |
| Linoleic acid* | 0.007 | ↑ |
| Valine* | 0.014 | ↑ |
| Hypoxanthine* | 0.004 | ↑ |
| No ID (RI:1530)* | 0.015 | ↑ |
| Alcohol (RI:1696)* | 0.002 | ↑ |
| Inorganic compound (RI:1573) | 0.004 | ↑ |
| Tyrosine* | 0.005 | ↑ |
| Serine* | 0.007 | ↑ |
| Inosine* | 0.007 | ↑ |
| Hexadecanoic acid* | 0.002 | ↑ |
| Glutamine* | 0.016 | ↑ |
| Carbohydrate and Carbohydrate conjugate (RI:1823)* | 0.004 | ↑ |
| Cysteine* | 0.003 | ↑ |
| Malic acid* | 0.003 | ↑ |
| No ID (RI:1538) | 0.003 | ↑ |
| Amino acid and Amino Acid conjugate (RI:1295) | 0.010 | ↑ |
| Phosphoethanolamine | 0.025 | ↑ |
| Threonine* | 0.007 | ↑ |
| Carbohydrate and Carbohydrate conjugate (RI:1834)* | 0.002 | ↑ |
| Carbohydrate and Carbohydrate conjugate (RI:2129) | 0.013 | ↑ |
| No ID (RI:3378) | 0.007 | ↑ |
| No ID (RI:3505)* | 0.013 | ↑ |
| Adenosine* | 0.005 | ↑ |
| No ID (RI:2014)* | 0.003 | ↑ |
| Organic acid (RI:1178) | 0.013 | ↑ |
| Lysine* | 0.013 | ↑ |
| Phenylalanine* | 0.053 | ↑ |
| Ornithine* | 0.007 | ↑ |
| Glyceric acid-3-phosphate* | 0.055 | ↑ |
| No ID (RI:1812)* | 0.004 | ↑ |
| Glycerol-3-phosphate | 0.032 | ↑ |
| No ID (RI:1725)* | 0.053 | ↑ |
| Stearic acid | 0.020 | ↑ |
| Oleic acid* | 0.003 | ↑ |
| scyllo-Inositol* | 0.014 | ↑ |
| No ID (RI:2166) | 0.045 | ↓ |
| Aspartic acid | 0.010 | ↑ |
| Leucine* | 0.014 | ↑ |
| No ID (RI:1506) | 0.063 | ↓ |
| Asparagine | 0.007 | ↑ |
| No ID (RI:2455) | 0.028 | ↑ |
| No ID (RI:1560) | 0.005 | ↑ |
| Dehydroascorbic acid*† | 0.010 | ↑ |
| Cystine* | 0.013 | ↑ |
| No ID (RI:3470) | 0.126 | ↓ |
| Citric acid* | 0.001 | ↑ |
| No ID (RI:1738) | 0.020 | ↓ |
| No ID (RI:2146) | 0.003 | ↓ |
| Fructose-6-phosphate | 0.034 | ↑ |
| No ID (RI:1217)* | 0.010 | ↑ |
| Taurine | 0.039 | ↑ |
| 2-Aminoadipic acid* | 0.025 | ↑ |
| myo-Inositol* | 0.039 | ↑ |
| Creatinine* | 0.039 | ↑ |

Significant changes defined as VIP > 0.9 in OPLS-DA or *P* < 0.05, Mann Whitney U-test, indicated with arrow. RI = Retention Index.

* Significantly differentiating metabolites (*P* < 0.05, Mann Whitney U-test, or VIP > 0.9) between PCa bone metastases and bone metastases from other cancers; breast, kidney, and squamous cancer (BCa, KCa and SCa, Table S4). †Can originate not only from Dehydroasorbic acid but also from Ascorbic acid.
