# Supplementary material for: Metabolomic Characterization of Human Prostate Cancer Bone Metastases Reveals Increased Levels of Cholesterol
Source: PLoS One. 2010 Dec 3;5(12):e14175. doi: 10.1371/journal.pone.0014175 (PMC2997052; doi:10.1371/journal.pone.0014175)
Supplement: Table S3 — (0.04 MB DOC) [file pone.0014175.s004.doc]

**Table S3.** The top 15 canonical pathways listed by Ingenuity pathway analysis for prostate cancer bone metastases

| **Canonical Pathways** | **Molecules**^†^ |
| --- | --- |
| Aminoacyl-tRNA Biosynthesis | L-valine, L-phenylalanine, L-glutamic acid, L-glutamine, L-threonine, L-asparagine, glycine, L-serine, L-aspartic acid, L-tyrosine, L-cysteine, L-lysine |
| Nitrogen Metabolism | L-phenylalanine, L-glutamic acid, L-glutamine, L-asparagine, glycine, L-aspartic acid, L-tyrosine, taurine |
| Cyanoamino Acid Metabolism | L-glutamic acid, L-asparagine, glycine, L-serine, L-aspartic acid, L-cysteine |
| Glycine, Serine and Threonine Metabolism | L-threonine, glycine, L-serine, L-aspartic acid, L-cysteine |
| Urea Cycle and Metabolism of Amino Groups | L-glutamic acid, fumaric acid, L-aspartic acid, L-ornithine |
| Glutamate Metabolism | L-glutamic acid, L-glutamine, fumaric acid, citric acid |
| Alanine and Aspartate Metabolism | L-asparagine, fumaric acid, L-aspartic acid, citric acid |
| Lysine Biosynthesis | 2-aminoadipic acid, L-aspartic acid, L-lysine |
| Glutathione Metabolism | L-glutamic acid, pyrrolidonecarboxylic acid, glycine, L-cysteine |
| Arginine and Proline Metabolism | L-glutamic acid, fumaric acid, L-aspartic acid, L-ornithine |
| D-glutamine and D-glutamate Metabolism | L-glutamic acid, L-glutamine |
| Glutamate Receptor Signaling | L-glutamic acid, L-glutamine, glycine |
| Bile Acid Biosynthesis | cholesterol, glycine, taurine |
| Taurine and Hypotaurine Metabolism | taurine, L-cysteine |
| Phenylalanine, Tyrosine and Tryptophan  Biosynthesis | L-phenylalanine, L-tyrosine |

^†^Included in pathway analysis were metabolites that were significantly changed in prostate cancer bone metastases as compared to normal bone (Table S1)
