# Supplementary material for: Metabolomic Characterization of Human Prostate Cancer Bone Metastases Reveals Increased Levels of Cholesterol
Source: PLoS One. 2010 Dec 3;5(12):e14175. doi: 10.1371/journal.pone.0014175 (PMC2997052; doi:10.1371/journal.pone.0014175)
Supplement: Table S4 — (0.03 MB DOC) [file pone.0014175.s005.doc]

**Table S4.** The top five molecular and cellular functions listed by Ingenuity pathway analysis for prostate cancer bone metastases

| **Molecular and cellular functions** | **Molecules** |
| --- | --- |
| Amino Acid Metabolism | L-phenylalanine, glycine, adenosine, L-cysteine, cholesterol, L-glutamic acid, L-threonine, 2-aminoadipic acid, L-serine, L-aspartic acid, L-tyrosine, taurine, L-ornithine, L-lysine |
| Molecular Transport | L-phenylalanine, adenosine, glycine, citric acid, L-cysteine, linoleic acid, cholesterol, L-glutamic acid, stearic acid, L-threonine, 2-aminoadipic acid, arachidonic acid, L-serine, L-aspartic acid, L-ornithine, L-tyrosine, taurine, L-lysine |
| Small Molecule Biochemistry | L-phenylalanine, adenosine, glycine, citric acid, L-cysteine, linoleic acid, cholesterol, inosine, uridine, L-glutamic acid, stearic acid, L-threonine, 2-aminoadipic acid, arachidonic acid, L-serine, L-aspartic acid, L-ornithine, L-tyrosine, taurine, glycerophosphoric acid, L-lysine |
| Cell Cycle | linoleic acid, L-glutamic acid, stearic acid, L-asparagine, glycine, adenosine, arachidonic acid, L-aspartic acid, L-serine, L-ornithine |
| Carbohydrate Metabolism | linoleic acid, cholesterol, uridine, L-phenylalanine, stearic acid, L-threonine, adenosine, arachidonic acid, citric acid, L-tyrosine, L-lysine, L-cysteine |

^†^Included in pathway analysis were metabolites that were significantly changed in prostate cancer bone metastases as compared to normal bone (Table S1)
