# Supplementary material for: Metabolomic Characterization of Human Prostate Cancer Bone Metastases Reveals Increased Levels of Cholesterol
Source: PLoS One. 2010 Dec 3;5(12):e14175. doi: 10.1371/journal.pone.0014175 (PMC2997052; doi:10.1371/journal.pone.0014175)
Supplement: Table S5 — (0.07 MB DOC) [file pone.0014175.s006.doc]

**Table S5.** Significantly differentiating metabolites between prostate cancer (PCa) bone metastases and bone metastases from other cancers.

| **Metabolite** | **p-value** | **Increase/Decrease**  **PCa vs. BCa, KCa, and SCa**† |
| --- | --- | --- |
| Cholesterol | <0.001 | ↑ |
| Fumaric acid | 0.001 | ↑ |
| Threonic acid | 0.001 | ↑ |
| Serine | 0.003 | ↑ |
| myo-Inositol | 0.002 | ↑ |
| Phenylalanine | 0.001 | ↑ |
| Tyrosine | 0.002 | ↑ |
| Threonine | 0.003 | ↑ |
| Creatinine | 0.001 | ↑ |
| No ID (RI:3033.3) | 0.002 | ↑ |
| Carbohydrate and Carbohydrate conjugate (RI:1823) | 0.004 | ↑ |
| Hypoxanthine | 0.009 | ↑ |
| Linoleic acid | 0.008 | ↑ |
| Inosine | 0.021 | ↑ |
| Alcohols (RI:1696) | 0.005 | ↑ |
| Lysine | 0.006 | ↑ |
| Malic acid | 0.004 | ↑ |
| No ID (RI:3505) | 0.003 | ↑ |
| Valine | 0.006 | ↑ |
| No ID (RI:1482) | 0.013 | ↑ |
| No ID (RI:1812) | 0.004 | ↑ |
| Adenosine | 0.014 | ↑ |
| Cysteine | 0.004 | ↑ |
| No ID (RI:3701) | 0.005 | ↑ |
| Inositol | 0.006 | ↑ |
| Citric acid | <0.001 | ↑ |
| No ID (RI:1506) | 0.026 | ↓ |
| No ID (RI:2014) | 0.039 | ↑ |
| No ID (RI:1596) | 0.005 | ↑ |
| Uridine | 0.062 | ↑ |
| Pyroglutamic acid | 0.042 | ↑ |
| Cystine | 0.072 | ↑ |
| myo-Inositol-1-phosphate | 0.033 | ↑ |
| Glutamine | 0.021 | ↑ |
| No ID (RI:3602) | 0.014 | ↑ |
| Ornithine | 0.014 | ↑ |
| Guanine | 0.060 | ↑ |
| Nucleoside and Nucleoside conjugate (RI:2817) | 0.051 | ↑ |
| No ID (3.RI:2002) | 0.013 | ↑ |
| No ID (RI:2203) | 0.016 | ↑ |
| No ID (RI:1217) | 0.048 | ↑ |
| Carbohydrate and Carbohydrate conjugate (RI:3531) | 0.239 | ↓ |
| Glyceric acid-3-phosphate | 0.228 | ↑ |
| Carbohydrate and Carbohydrate conjugate (RI:1834) | 0.033 | ↑ |
| No ID (RI:3006) | 0.020 | ↑ |
| No ID (RI:2144) | 0.060 | ↑ |
| Hexadecanoic acid | 0.033 | ↑ |
| Glutamic acid | 0.039 | ↑ |
| Glyceric acid | 0.058 | ↑ |
| alpha-Tocopherol | 0.121 | ↑ |
| Oleic acid | 0.033 | ↑ |
| 2-Aminoadipic acid | 0.006 | ↑ |
| Organic acid (RI:1362) | 0.076 | ↑ |
| No ID (RI:1235) | 0.086 | ↓ |
| No ID (RI:1342) | 0.069 | ↓ |
| No ID (RI:1382) | 0.006 | ↑ |
| Phosphoric acid | 0.137 | ↑ |
| Leucine | 0.248 | ↑ |
| No ID (RI:1675) | 0.026 | ↑ |
| Urea | 0.029 | ↑ |
| No ID (RI:1530) | 0.033 | ↑ |
| No ID (RI:1725) | 0.048 | ↑ |

Significant changes defined as VIP > 0.9 in OPLS-DA or *P* < 0.05, Mann Whitney U-test, indicated with arrow. RI = Retention Index. †Breast, kidney, and squamous cancer (BCa, KCa, SCa)
