# Supplementary material for: Metabolomic Characterization of Human Prostate Cancer Bone Metastases Reveals Increased Levels of Cholesterol
Source: PLoS One. 2010 Dec 3;5(12):e14175. doi: 10.1371/journal.pone.0014175 (PMC2997052; doi:10.1371/journal.pone.0014175)
Supplement: Table S6 — (0.07 MB DOC) [file pone.0014175.s007.doc]

**Table S6.** Significantly differentiating metabolites between prostate tissue samples from prostate cancer patients diagnosed with bone metastases (M1) and patients with benign disease.

| **Metabolite name** | **p-value** | **Increase/Decrease in M1 vs. Benign** |
| --- | --- | --- |
| Fumaric acid* | 0.003 | ↑ |
| Phosphoryethanolamine | 0.003 | ↑ |
| Phosphoric acid | 0.001 | ↑ |
| Malic acid* | 0.003 | ↑ |
| Amino acid and Amino Acid conjugate (RI:1426) | 0.003 | ↑ |
| Inorganic compound (RI:1574) | 0.003 | ↑ |
| Glycerol-3-phosphate | 0.013 | ↑ |
| Dehydroascorbic acid *† | 0.004 | ↑ |
| No ID (RI:1201)* | 0.003 | ↑ |
| No ID (RI:1559) | 0.008 | ↑ |
| Carbohydrate and Carbohydrate conjugate (RI:1451) | 0.008 | ↑ |
| Glutamic acid | 0.008 | ↑ |
| No (RI:1208) | 0.015 | ↑ |
| Pyroglutamic acid | 0.004 | ↑ |
| Urea* | 0.011 | ↑ |
| Glycine | 0.011 | ↑ |
| No ID (RI:1467) | 0.011 | ↑ |
| No ID (RI:2204) | 0.018 | ↑ |
| No ID (RI:1677)* | 0.01 | ↑ |
| No ID (RI:1950) | 0.015 | ↑ |
| No ID (RI:1472)* | 0.049 | ↑ |
| myo-Inositol-1-phosphate | 0.035 | ↑ |
| Nucleoside and Nucleoside conjugate (RI:1340) | 0.015 | ↑ |
| Nucleoside and Nucleoside conjugate (RI:2814) | 0.048 | ↑ |
| 2-Amino-adipic acid | 0.018 | ↑ |
| Glutamine | 0.085 | ↑ |
| Threonic acid | 0.037 | ↑ |
| No ID (RI:1548) | 0.004 | ↑ |
| Aspartic acid | 0.064 | ↑ |
| No ID (RI:1383) | 0.035 | ↑ |
| Threonine* | 0.064 | ↑ |
| No ID (RI:2298) | 0.028 | ↑ |
| Guanosine | 0.093 | ↑ |
| Taurine | 0.02 | ↑ |
| No ID (RI:1971) | 0.015 | ↑ |
| No ID (RI:2026)* | 0.011 | ↑ |
| Adenosine-5-monophosphate | 0.886 | ↑ |
| Valine | 0.105 | ↑ |
| Creatinine | 0.015 | ↑ |
| No ID (RI:2818) | 0.338 | ↑ |
| No ID (RI:2855)* | 0.093 | ↓ |
| Aspargine* | 0.105 | ↑ |
| No ID (RI:1207) | 0.247 | ↑ |
| No ID (RI:1208) | 0.083 | ↑ |
| No ID (RI:2238) | 0.003 | ↑ |
| Hypoxanthine* | 0.048 | ↑ |
| Arachidonic acid | 0.083 | ↓ |
| Lysine | 0.083 | ↑ |
| Linoleic acid* | 0.11 | ↑ |
| No ID (RI:3860) | 0.277 | ↑ |
| No ID (RI:2265) | 0.083 | ↑ |
| No ID (RI:1731) | 0.565 | ↑ |
| No ID (RI:1399) | 0.225 | ↑ |
| No ID (RI:2471) | 0.247 | ↑ |
| No ID (RI:2372) | 0.105 | ↑ |
| No ID (RI:1363) | 0.565 | ↑ |
| No ID (RI:1533) | 0.021 | ↑ |
| No ID (RI:1989) | 0.132 | ↑ |
| No ID (RI:3569) | 0.132 | ↑ |
| No ID (RI:2311) | 0.037 | ↑ |
| Ethanolamine | 0.355 | ↓ |
| Cholesterol | 0.247 | ↑ |
| No ID (RI:1414) | 0.064 | ↑ |
| No ID (RI:3007) | 0.203 | ↑ |

Significant changes defined as VIP > 0.9 in OPLS-DA or *P* < 0.05, Mann Whitney U-test, indicated with arrow. RI = Retention Index.

*Significant changed metabolites also in comparison of prostate cancer tissue samples from high-risk patients with and without diagnosed bone metastases (Table S7). †Can originate not only from Dehydroasorbic acid but also from Ascorbic acid.
