# Supplementary material for: Metabolomic Characterization of Human Prostate Cancer Bone Metastases Reveals Increased Levels of Cholesterol
Source: PLoS One. 2010 Dec 3;5(12):e14175. doi: 10.1371/journal.pone.0014175 (PMC2997052; doi:10.1371/journal.pone.0014175)
Supplement: Table S7 — (0.07 MB DOC) [file pone.0014175.s008.doc]

**Table S7.** Significantly differentiating metabolites between prostate cancer tissue samples from high-risk patients with (M1) and without (M0) diagnosed bone metastases.

| **Metabolite** | **p-value** | **Increase/Decrease in M1 vs. M0** |
| --- | --- | --- |
| Nonanoic acid | 0.022 | ↓ |
| No ID (RI:3732) | 0.005 | ↓ |
| Aspargine | 0.003 | ↑ |
| No ID (RI:2632) | 0.02 | ↓ |
| No ID (RI:1367) | 0.015 | ↓ |
| No ID (RI:1206) | 0.049 | ↑ |
| Organic acid (RI:1162) | 0.021 | ↓ |
| No ID (RI:1419) | 0.011 | ↓ |
| No ID (RI:1227) | 0.028 | ↓ |
| Phenylalanine | 0.005 | ↑ |
| Oleic acid | 0.01 | ↑ |
| No ID (RI:1580) | 0.021 | ↓ |
| No ID (RI:3416) | 0.063 | ↑ |
| No ID (RI:1399) | 0.022 | ↓ |
| No ID (RI:1414) | 0.015 | ↓ |
| Tyrosine | 0.021 | ↑ |
| No ID (RI:3758) | 0.203 | ↑ |
| No ID (RI:2269) | 0.165 | ↓ |
| No ID (RI:1978) | 0.071 | ↓ |
| No ID (RI:2040) | 0.064 | ↓ |
| Fumaric acid | 0.004 | ↑ |
| No ID (RI:2471) | 0.121 | ↓ |
| Hypoxanthine | 0.004 | ↑ |
| No ID (RI:1731) | 0.035 | ↓ |
| No ID (RI:1732) | 0.085 | ↓ |
| No ID (RI:2026) | 0.039 | ↑ |
| No ID (RI:3492) | 0.062 | ↓ |
| Sterol (RI:2862) | 0.005 | ↓ |
| Adenosine | 0.302 | ↓ |
| Glyceric acid | 0.015 | ↑ |
| No ID (RI:3569) | 0.132 | ↓ |
| No ID (RI:2729) | 0.298 | ↓ |
| No ID (RI:1203) | 0.049 | ↓ |
| Alcohols (RI:1238) | 0.156 | ↓ |
| No ID (RI:1858) | 0.197 | ↓ |
| Dehydroascorbic acid† | 0.014 | ↑ |
| No ID (RI:2018) | 0.121 | ↓ |
| Organic acid (RI:1152) | 0.298 | ↓ |
| Amine (RI:1729) | 0.048 | ↓ |
| No ID (RI:1200.8) | 0.021 | ↑ |
| No ID (RI:3702) | 0.247 | ↓ |
| No ID (RI:1343) | 0.165 | ↓ |
| Carbohydrate and Carbohydrate conjugate (RI:2308) | 0.081 | ↓ |
| Organic acid (RI:2066) | 0.18 | ↓ |
| Glucose-6-phosphate | 0.083 | ↑ |
| Serine | 0.022 | ↑ |
| No ID (RI:1337) | 0.247 | ↓ |
| No ID (RI:1333) | 0.165 | ↑ |
| Guanosine | 0.203 | ↓ |
| No ID (RI:2496) | 0.245 | ↓ |
| Linoleic acid | 0.01 | ↑ |
| Itaconic acid | 0.355 | ↓ |
| No ID (RI:2855) | 0.105 | ↓ |
| Urea | 0.037 | ↑ |
| Threonine | 0.032 | ↑ |
| Carbohydrate and Carbohydrate conjugate (RI:1451) | 0.165 | ↓ |
| Carbohydrate and Carbohydrate conjugate (RI:2297) | 0.083 | ↑ |
| No ID (RI:1472) | 0.049 | ↑ |
| No ID (RI:1210) | 0.355 | ↑ |
| Glutaric acid | 0.132 | ↓ |
| No ID (RI:2372) | 0.156 | ↓ |
| Malic acid | 0.01 | ↑ |
| No ID (RI:1677) | 0.015 | ↑ |
| Cystine | 0.028 | ↑ |

Significant changes defined as VIP > 0.9 in OPLS-DA or *P* < 0.05, Mann Whitney U-test, indicated with arrow. RI = Retention Index. †Can originate not only from Dehydroasorbic acid but also from Ascorbic acid.
