# Supplementary material for: Metabolomic Characterization of Human Prostate Cancer Bone Metastases Reveals Increased Levels of Cholesterol
Source: PLoS One. 2010 Dec 3;5(12):e14175. doi: 10.1371/journal.pone.0014175 (PMC2997052; doi:10.1371/journal.pone.0014175)
Supplement: Table S8 — (0.06 MB DOC) [file pone.0014175.s009.doc]

**Table S8.** Significantly differentiating metabolites between blood plasma samples from prostate cancer patients diagnosed with bone metastases (M1) and patients with benign disease.

| **Metabolite** | **p-value** | **Increase/Decrease in M1 vs. Benign** |
| --- | --- | --- |
| No ID (RI:1430)* | 0.021 | ↓ |
| No ID (RI:1420)* | 0.018 | ↓ |
| No ID (RI:2104) | 0.174 | ↓ |
| No ID (RI:1204)* | 0.036 | ↑ |
| No ID (RI:1464) | 0.118 | ↓ |
| No ID (RI:1388)* | 0.058 | ↑ |
| No ID (RI:1734)* | 0.029 | ↑ |
| Phenylalanine* | 0.065 | ↑ |
| No ID (RI:3000) | 0.056 | ↑ |
| No ID (RI:1428) | 0.056 | ↑ |
| No ID (RI:2168) | 0.071 | ↓ |
| No ID (RI:1542)* | 0.111 | ↓ |
| No ID (RI:1307)* | 0.145 | ↓ |
| No ID (RI:1490)* | 0.354 | ↓ |
| No ID (RI:1375)* | 0.058 | ↓ |
| Organic acid (RI:1324)* | 0.264 | ↓ |
| No ID (RI:2049)* | 0.144 | ↑ |
| Taurine* | 0.069 | ↑ |
| No ID (RI:1498)* | 0.172 | ↑ |
| No ID (RI:1946) | 0.081 | ↑ |
| Pseudouridine* | 0.036 | ↑ |
| No ID (RI:1331) | 0.102 | ↓ |
| Organic acid (RI:1534)* | 0.051 | ↑ |
| No ID (RI:2081) | 0.026 | ↓ |
| Alcohols (RI:1275) | 0.093 | ↓ |
| No ID (RI:1741) | 0.133 | ↑ |
| No ID (RI:1695)* | 0.174 | ↑ |
| Cystine | 0.093 | ↑ |
| Glutamic acid* | 0.134 | ↑ |
| No ID (RI:1338)* | 0.147 | ↓ |
| Sterol (RI:2862) | 0.328 | ↑ |
| No ID (RI:1842) | 0.157 | ↓ |
| No ID (RI:1574) | 0.355 | ↑ |
| No ID (RI:1317)* | 0.099 | ↓ |
| Stearic acid* | 0.093 | ↓ |
| Creatinine* | 0.237 | ↑ |
| No ID (RI:1839)* | 0.172 | ↑ |
| Organic acid (RI:2139)* | 0.125 | ↓ |
| No ID (RI:1197)* | 0.369 | ↑ |
| No ID (RI:2163) | 0.409 | ↓ |
| Glucose* | 0.272 | ↑ |
| Amino acid and Amino Acid conjugate (RI:1519.9)* | 0.057 | ↑ |

Significant changes defined as VIP > 0.9 in OPLS-DA or *P* < 0.05, Mann Whitney U-test, indicated with arrow. RI = Retention Index. *Significantly changed metabolites also in comparison of plasma samples from high-risk patients with and without diagnosed bone metastases (Table S9).
