# Supplementary material for: Metabolomic Characterization of Human Prostate Cancer Bone Metastases Reveals Increased Levels of Cholesterol
Source: PLoS One. 2010 Dec 3;5(12):e14175. doi: 10.1371/journal.pone.0014175 (PMC2997052; doi:10.1371/journal.pone.0014175)
Supplement: Table S9 — (0.07 MB DOC) [file pone.0014175.s010.doc]

**Table S9.** Significant changed metabolites between blood plasma samples from high-risk prostate cancer patients with (M1) and without (M0) diagnosed bone metastases.

| **Metabolite** | **p-value** | **Increase/Decrease in M1 vs. M0** |
| --- | --- | --- |
| Amino acid and Amino Acid conjugate (RI:1375) | 0.009 | ↓ |
| Phenylalanine | 0.013 | ↑ |
| Pyroglutamic acid | 0.009 | ↑ |
| No ID (RI:1388) | 0.017 | ↑ |
| No ID (RI:1695) | 0.01 | ↑ |
| No ID (RI:1204) | 0.021 | ↑ |
| myo-inositol | 0.174 | ↑ |
| No ID (RI:1317) | 0.024 | ↓ |
| No ID (RI:1307) | 0.039 | ↓ |
| No ID (RI:1498) | 0.023 | ↑ |
| No ID (RI:1375) | 0.031 | ↓ |
| No ID (RI:1420) | 0.032 | ↓ |
| No ID (RI:2420) | 0.12 | ↑ |
| No ID (RI:2014) | 0.089 | ↑ |
| No ID (RI:1734) | 0.039 | ↑ |
| 2-Methyl-3-hydroxybutyric acid | 0.447 | ↓ |
| Methionine | 0.05 | ↑ |
| No ID (RI:2052) | 0.05 | ↓ |
| No ID (RI:1146) | 0.033 | ↓ |
| No ID (RI:2455) | 0.052 | ↓ |
| No ID (RI:1542) | 0.118 | ↓ |
| Tyrosine | 0.108 | ↑ |
| Organic acid (RI:1324) | 0.061 | ↓ |
| Glutamic acid | 0.15 | ↑ |
| No ID (RI:1430) | 0.067 | ↓ |
| No ID (RI:1545) | 0.048 | ↑ |
| Isoleucine | 0.232 | ↑ |
| No ID (RI:2123) | 0.19 | ↑ |
| No ID (RI:2299) | 0.04 | ↑ |
| Organic acid (RI:1534) | 0.05 | ↑ |
| No ID (RI:1839) | 0.106 | ↑ |
| No ID (RI:1452) | 0.055 | ↑ |
| Glycine | 0.141 | ↑ |
| alpha-Tocopherol | 0.138 | ↑ |
| No ID (RI:1423) | 0.061 | ↓ |
| Alcohol (RI:1705) | 0.129 | ↑ |
| Palmitelaidic acid | 0.256 | ↓ |
| myo-inositol-1-phosphate | 0.176 | ↑ |
| Organic acid (RI:2139) | 0.168 | ↓ |
| Stearic acid | 0.19 | ↓ |
| No ID (RI:2793) | 0.176 | ↑ |
| No ID (RI:1338) | 0.127 | ↓ |
| Creatinine | 0.168 | ↑ |
| Hexadecanoic acid | 0.275 | ↓ |
| No ID (RI:1447) | 0.089 | ↓ |
| Oleic acid | 0.19 | ↓ |
| No ID (RI:2049) | 0.244 | ↑ |
| Taurine | 0.222 | ↑ |
| No ID (RI:1197) | 0.275 | ↑ |
| No ID (RI:2419) | 0.308 | ↑ |
| Amino acid and Amino Acid conjugate (RI:1519.9) | 0.337 | ↑ |
| No ID (RI:2165) | 0.198 | ↓ |
| No ID (RI:1566) | 0.118 | ↑ |
| Tryptophan | 0.15 | ↑ |
| Pseudouridine | 0.089 | ↑ |
| Valine | 0.335 | ↑ |
| No ID (RI:1560) | 0.168 | ↑ |
| Threonic acid | 0.251 | ↑ |
| Glucose | 0.206 | ↑ |
| Ornithine | 0.174 | ↑ |
| No ID (RI:3546) | 0.264 | ↓ |
| Fructose | 0.222 | ↑ |
| No ID (RI:1560) | 0.089 | ↓ |
| No ID (RI:2376) | 0.106 | ↑ |
| No ID (RI:1490) | 0.036 | ↑ |

Significant changes defined as VIP > 0.9 in OPLS-DA or *P* < 0.05, Mann Whitney U-test, indicated with arrow. RI = Retention Index.
