# Supplementary material for: Metabolomic Characterization of Human Prostate Cancer Bone Metastases Reveals Increased Levels of Cholesterol
Source: PLoS One. 2010 Dec 3;5(12):e14175. doi: 10.1371/journal.pone.0014175 (PMC2997052; doi:10.1371/journal.pone.0014175)
Supplement: Table S10 — (0.10 MB DOC) [file pone.0014175.s011.doc]

**Table S10**. Summary of identified metabolites in bone tissue, prostate tissue and plasma.

| **Metabolite name** | **Bone tissue** | **Prostate tissue** | **Plasma** |  |
| --- | --- | --- | --- | --- |
| 2-Amino-adipic acid | + | + | + |  |
| 2-Methyl-3-hydroxybutyric acid |  |  | + |  |
| 3-amino-2-methylpropanoic acid | + |  |  |  |
| Adenosine | + | + |  |  |
| Adenosine-5-monophosphate | + | + |  |  |
| Alanine | + |  |  |  |
| Aminomalonic acid |  |  | + |  |
| Arachidonic acid | + | + | + |  |
| Asparagine | + | + | + |  |
| Aspartic acid | + | + | + |  |
| Campesterol |  |  | + |  |
| Cholesterol | + | + | + |  |
| Citric acid | + | + | + |  |
| Creatinine | + | + | + |  |
| Cysteine | + | + | + |  |
| Cystine | + | + | + |  |
| Dehydroascorbic acid dimer | + | + |  |  |
| Docosahexaenoic acid |  | + | + |  |
| Elaidic acid |  | + |  |  |
| Ethanolamine |  | + |  |  |
| Fructose |  |  | + |  |
| Fumaric acid | + | + |  |  |
| Glucose | + | + | + |  |
| Glucose-6-phosphate | + | + |  |  |
| Glutamic acid | + | + | + |  |
| Glutamine | + | + | + |  |
| Glutaric acid | + | + |  |  |
| Glyceric acid | + | + | + |  |
| Glyceric acid-3-phosphate | + | + |  |  |
| Glycerol-3-phosphate | + | + |  |  |
| Glycine | + | + | + |  |
| Guanosine | + | + |  |  |
| Hexadecanoic acid | + | + | + |  |
| Hypoxanthine | + | + |  |  |
| Indole-3-acetic acid |  |  | + |  |
| Inosine | + | + |  |  |
| Itaconic acid | + | + |  |  |
| Lauric acid |  |  | + |  |
| Linoleic acid | + | + | + |  |
| Lysine | + | + | + |  |
| Malic acid | + | + |  |  |
| Methionine | + | + | + |  |
| Myo-inositol | + | + | + |  |
| Myo-inositol-1-phosphate | + | + | + |  |
| Nonanoic acid | + | + | + |  |
| Oleic acid | + | + | + |  |
| Ornithine | + | + | + |  |
| Palmitelaidic acid |  |  | + |  |
| Phenylalanine | + | + | + |  |
| Phosphoric acid | + | + | + |  |
| Phosphoryethanolamine | + | + |  |  |
| Proline | + |  | + |  |
| Pseudouridine |  |  | + |  |
| Putrescine |  | + |  |  |
| Pyroglutamic acid | + | + | + |  |
| Salicylic acid |  |  | + |  |
| Scyllo-inositol | + | + |  |  |
| Serine | + | + | + |  |
| Spermidine | + | + |  |  |
| Spermine |  | + |  |  |
| Squalene | + |  |  |  |
| Stearic acid | + | + | + |  |
| Succinic acid | + | + | + |  |
| Tartaric acid |  | + |  |  |
| Taurine | + | + | + |  |
| Tetradecanoic acid |  |  | + |  |
| Threonic acid | + | + | + |  |
| Threonine | + | + | + |  |
| alfa-Tocopherol |  |  | + |  |
| beta-Tocopherol | + |  |  |  |
| Tryptophan |  |  | + |  |
| Tyrosine | + | + | + |  |
| Uracil | + | + |  |  |
| Urea | + | + | + |  |
| Uric acid | + |  | + |  |
| Uridine | + | + |  |  |
| Valine | + | + | + |  |

+Detected and identified metabolite.
