# Supplementary figures and images for: Metabolomic Characterization of Human Prostate Cancer Bone Metastases Reveals Increased Levels of Cholesterol
Source: PLoS One. 2010 Dec 3;5(12):e14175. doi: 10.1371/journal.pone.0014175 (PMC2997052; doi:10.1371/journal.pone.0014175)

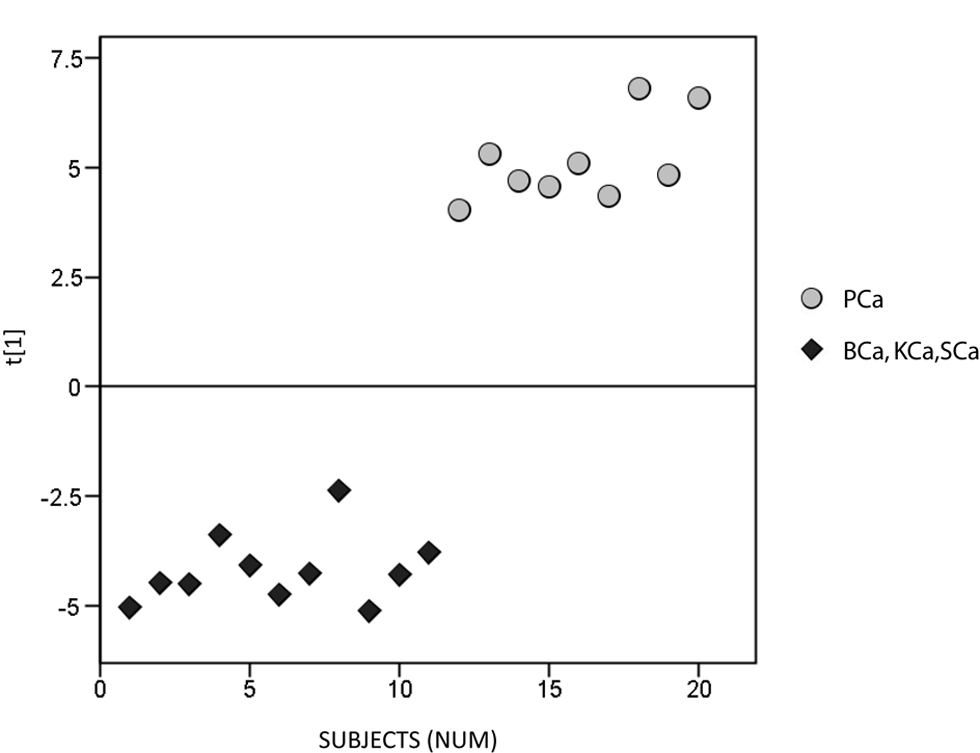

Supplement: Figure S1 — Multivariate modelling in the search for a unique metabolite pattern in prostate cancer (PCa) bone metastases. OPLS-DA score vector (t[1]) showing a clear and significant difference between PCa bone metastases and bone metastases from other cancers: breast, kidney, and squamous cancer (BCa, KCa and SCa). (2.24 MB TIF) [file pone.0014175.s012.tif]

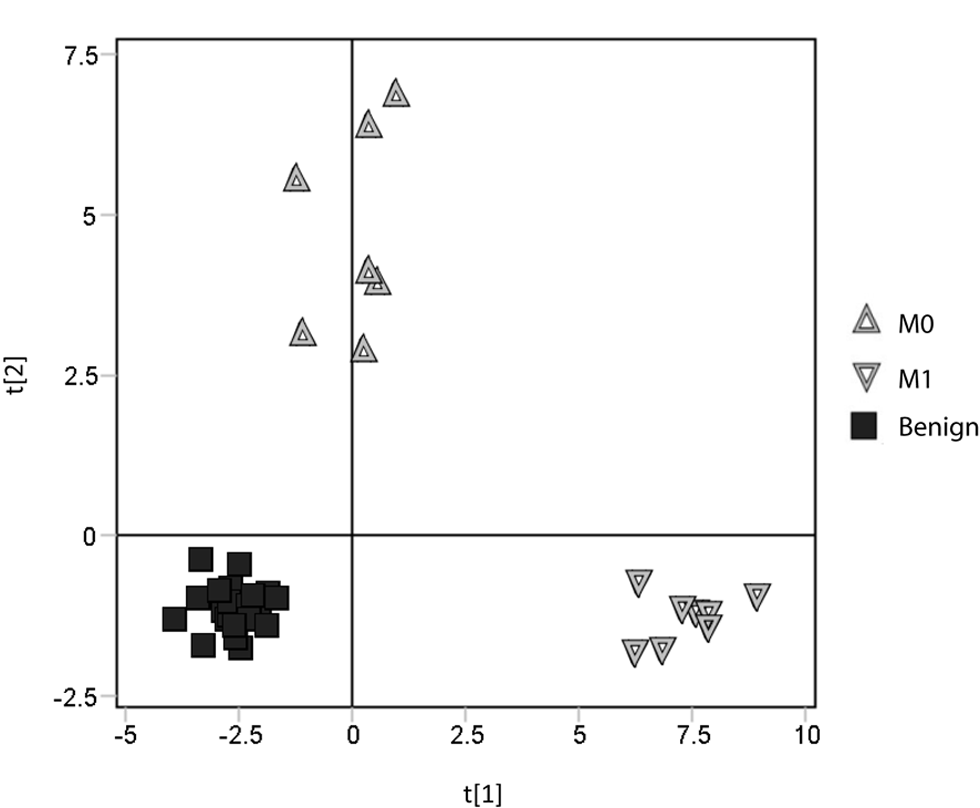

Supplement: Figure S2 — Metabolomic differences between primary prostate cancer tissues from high-risk patients with (M1) and without (M0) established bone metastases. OPLS-DA score plot (t[2]/t[1]) revealing clear differences in metabolic signatures in primary prostate tumor tissue from patients with and without diagnosed bone metastases compared to benign prostate tissue. (2.40 MB TIF) [file pone.0014175.s013.tif]

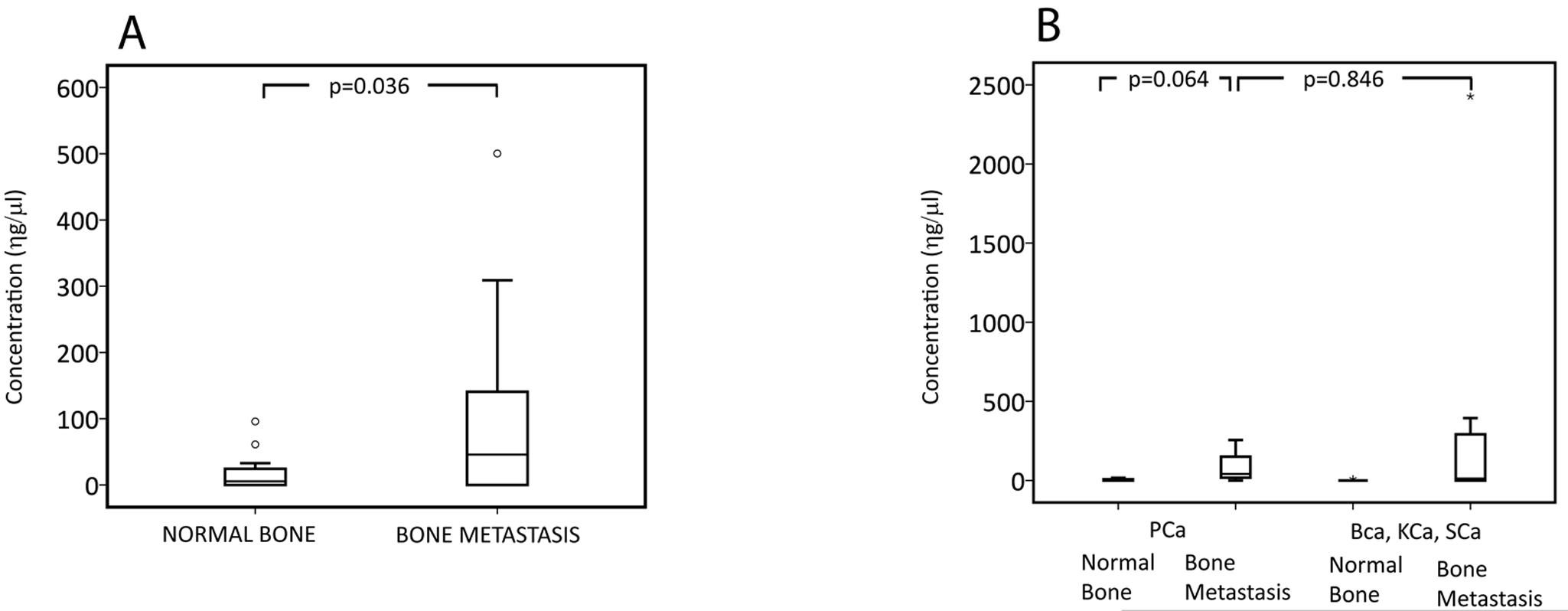

Supplement: Figure S3 — Sarcosine levels in bone metastases. A. Box plot for sarcosine concentration showing significantly higher levels in prostate cancer (PCa) bone metastases compared to normal bone. B. Box plot for sarcosine in test set showing higher levels in PCa bone metastases compared to normal bone but no difference in levels in PCa bone metastases compared to bone metastases from other cancers; breast, kidney, and squamous cancer (BCa, KCa and SCa). (4.93 MB TIF) [file pone.0014175.s014.tif]
